# Keystone active bacterial lineages associated with *Penaeus stylirostris* shrimp health across larvae stages

**DOI:** 10.1371/journal.pone.0335417

**Published:** 2025-10-29

**Authors:** Nolwenn Callac, Nelly Wabete, Carolane Giraud, Valérie Perez, Dominique Ansquer, Jean-Sébastien Lam, Dominique Pham, Viviane Boulo

**Affiliations:** 1 Ifremer, IRD, Université de la Nouvelle-Calédonie, Université de La Réunion, UMR ENTROPIE, Nouméa, New Caledonia; 2 Ifremer, MASAE Microbiologie Aliment Santé Environnement, Nantes, France; 3 Interactions Hôtes Pathogènes Environnements (IHPE), Université de Montpellier, CNRS, Ifremer, Université de Perpignan Via Domitia, Montpellier, France; Sao Paulo State University Julio de Mesquita Filho: Universidade Estadual Paulista Julio de Mesquita Filho, BRAZIL

## Abstract

The Pacific blue shrimp, *Penaeus stylirostris*, reared in New Caledonia, is economically important for the territory. However, since 2005, this sector has been facing huge larval mortalities occurring at all larval stages in hatcheries and for which no causes have yet been found. Microbial dysbiosis of the larvae are suspected as factors leading to larval death. To test this hypothesis, we monitored daily the larval health based on their survival rate and developmental stage and explored the active microbiota of the larvae by sequencing the V4 region of the 16S rRNA sequence. Richness proxies exhibited lower values in the unhealthy (high mortality rate) larval microbiota compared to the healthy one, highlighting a loss of microbial diversity in the unhealthy larvae. Venn diagram comparisons displayed specific taxa associated with a given larval stage and health with several taxa being vertically transmitted among multiple larval stages of a same health status as shown in the core microbiota. Besides, at the zoea stage, when the mortalities started, three ASVs related to *Tenacibaculum*, *Microscilla* and *Bernardetia* were specific of the unhealthy zoea suggesting that the zoea stage is crucial for dysbiosis induction. It is therefore probably at this stage that dysbiosis of the microbiota could evolve into larval pathobiome and lead to larval death. Thus, identifying specific lineages related to dysbiosis, being specific or correlated to unhealthy larvae or to the pathobiome, and demonstrating their pathogenicity, could ultimately support larval rearing by enabling targeted responses to mitigate their impact.

## Introduction

The development, growth, physiology, fitness, nutriment intake, or even health of various macroorganisms, is under the influence of microbial communities that they harbor and with which they are in close association to form the holobiont [1–3]. Typically, a holobiont comprises a eukaryotic species, the host, and its associated microbiota, also known as symbionts [1,3]. Together they become a functional entity; however, according to environmental conditions, the nature of the interactions between the host and its microbiota can change [4,5]. These interactions may shift from mutualistic symbiosis to other forms, including parasitism or detrimental associations. In such cases, the holobiont may transition into a pathobiont [6–8]. Indeed, modification of the healthy microbiota, caused by imbalance and disruption of its composition and loss of commensal and/or beneficial microorganisms, leads to dysbiosis, while pathobiont refers to microorganisms that are opportunists and can foster disease [6–8]. Host microbiota is therefore, one of the main cornerstones of the holobiont heath.

Aquatic animals live in water where they are constantly surrounded by water microbiota with some being primary producers of the food web, other being involved in many biogeochemical cycles, and some being involved in animal health being probiotic, predators or even pathogens [9–13]. In aquaculture, disease outbreaks frequently cause mass mortalities due to either a unique pathogen that can be a bacterium [14], a parasite [15], or a virus [16], or to multifactorial and polymicrobial causes that affect the holobiont, especially its microbiota [17,18]. In multifactorial and polymicrobial cases, dysbiosis could be due to the presence of a pathogen and/or to environmental pressures and stresses, that can turn the microbiome into a pathobiome with the prevalence of opportunistic bacteria with an r-strategy or pathogenic bacteria in the host microbiota [17]. Disease, dysbiosis induction and therefore the establishment of the pathobiome can be threatening for aquacultured species [17] resulting in significant mortalities along with important economic losses. It is therefore important to distinguish beneficial from unbeneficial microbiome in mariculture holobiont. In shrimp aquaculture, microbial biomarkers of diseased and healthy penaeids have been evidenced for adults and larvae *Penaeus vannamei*, but to date, we lack data about larval microbial lineages related to healthy and unhealthy *Penaeus stylirostris* larvae. Shrimp larvae are reared in hatcheries in tanks that contain seawater hosting complex microbial communities made of commensal, beneficial, neutral or even pathogenic bacteria which together maintain an appropriate balance of the rearing water microflora and chemical composition of the water (nutrient recycling, eutrophication, removal of certain molecules and pollutants), which are key parameters to successful rearings [19,20]. Therefore, microorganisms are a cornerstone component of the hatchery environment and are also able to shape their rearing ecosystem through various metabolic reactions such as organic matter degradation, denitrification or organic matter turnover. Moreover, these microorganisms can also be involved in the animal development, welfare, or nutrition intake through complex interactions between them, the larvae and their microbiota [19,20]. Environmental conditions of the hatchery (water quality, temperature, management practice) are known to influence and to shape microbial assembly through deterministic (non-random) processes, along with stochastic (random) processes [21,22]. Consequently, hatchery environment participates greatly in shaping host-microbiota interactions [20].

In New Caledonia, since 2005, the production of the Pacific blue shrimp *Penaeus stylirostris* has been facing issues in supplying farms with postlarvae due to a dramatic drop of the larval production in hatcheries. Mains consequences are the impairment of shrimp farming and the shrimp industry, and consequently the economy of the territory [23]. To date, even if numerous bacterial analyses have been performed by the Neo-Caledonian Network of Shrimp Epidemiological Vigilance (REC-DAVAR) at each larval mortality episode, no larval septicemia have been evidenced, therefore refuting the bacterial infection. In the same way, analysis to detect viruses responsible for the main shrimp infections worldwide [17,18], and the parasitic infection have been conducted, but no infection has been detected. Consequently, multifactorial causes seem to induce massive larval mortalities which often starts at the zoea stage [24,25]. Getting clues to understand the mortality process is essential to prevent such events and to develop tools to predict the fate of the larval rearing and perpetuate shrimp farming in New Caledonia. Indeed, with only 3 hatcheries in the territory selling postlarvae to 17 farms, care must be taken to ensure shrimp production and to maintain this economic source in New Caledonia. Our hypotheses were that dysbiosis could lead to a pathobiome that induces larval death, with specific lineages related to the unhealthy larvae that can be transmitted from one larval stage to another; and, that specific taxa that can be linked to a specific larval stage and health. To test these hypotheses, we analyzed data from different rearings using the same pool of eggs from the same breeders to overcome breeders’ influence. Our data revealed a loss of microbial diversity richness in the unhealthy larvae compared to the healthy one. We also evidenced lineages that were specific of a larval stage and health. Furthermore, at the zoea stage, when mortality started to occur, we found three ASVs affiliated to *Tenacibaculum*, *Microscilla* and *Bernardetia,* that were specific to unhealthy zoea. Some were affiliated to genera known to be found in the microbiota of diseased animals, suggesting their putative role in triggering dysbiosis.

## Materials and methods

### Hatchery experiment

Analyses were conducted using samples and data collected from rearings carried out in February 2019 in an experimental shrimp hatchery located in the Station Aquacole de Saint Vincent in Boulouparis, New Caledonia. All rearings used the same batch of eggs from a single group of breeders to eliminate breeder-related variability. Rearing tanks were filled with 150L of seawater that had been treated through different methods: Water T (treated water) or Water NT (non-treated water) (e.g., different filtrations processes and use or not skimmer or of UV chamber) as described in the S1Fig. Briefly, water T corresponds to seawater that was pumped from the lagoon to the primary reservoir and then passed through a sand filter and a membrane filter of 10 µm before being pulled into the secondary reservoir. Next, the water was continuously passed through a skimmer and a series of filters: 5 µm and 1 µm. Finally, the water circulated through a UV chamber before being added in the rearing tanks. Water NT corresponds to the seawater that was pumped from the lagoon to the primary reservoir and then passed through a sand filter and a membrane filter of 10 µm before being pulled into the secondary reservoir. Finally, before being added in the rearing tanks the water circulated through a 5 µm membrane filter. Each rearing condition was made in triplicate, and no water exchange occurred during the larval rearing. Erythromycin was added at 2 ppm on D0 and then on days D3, D5, D7 and D9, or on D3 and further on days D5, D7 and D9. The antibiotic (under veterinary advice) was added in three rearing conditions, for prophylactic purposes to prevent larval mortalities. *In fine,* five rearing water conditions were tested: 1) Water T without antibiotic named as TSA, 2) Water T with antibiotic first added on D3 named as TA3, 3) Water NT without antibiotic named as NTSA, 4) Water NT with antibiotic first added on D0 named as NTA0 and 5) Water NT with antibiotic first added on D3 named as NTA3.

**Fig 1:**
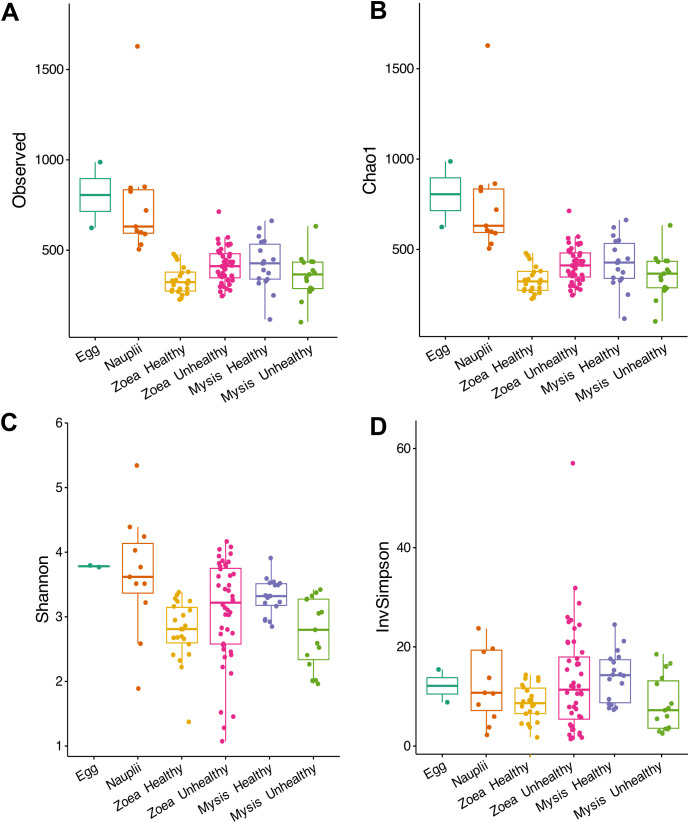
Alpha diversity indexes of the microbial diversity associated with the larvae. **(A)** Observed ASVs, **(B)** Chao1, **(C)** Shannon and **(D)** Inverse Simpson (InvSimpson). Data are available in S1 Table.[Subxref2] Larvae were classified as healthy if their survival rate was above or equal to the reference threshold (D0 = 100%, D1 between 95 and 100%, D2 ≥ 95%, D3 ≥ 90%, D4 ≥ 85%, D5 ≥ 80%, D6 ≥ 77%, D7 ≥ 75%, D8 ≥ 73% and D9 ≥ 70%); otherwise, they were considered as unhealthy.

The larvae were obtained by artificial inseminations of mature *Penaeus stylirostris* breeders as described by Pham et al. 2012 [26]. Then, after spawning and hatching, nauplii from the same reproduction day (D-1) were separated from egg debris and unhatched eggs, rinsed and pooled into a same batch to be further used for the experimentation. On the same day, D0, rearing tanks were filled with seawater treated as depicted in Fig 1. Also, on D0, ethylenediaminetetraacetic acid (EDTA) was added at 5 g.m^-3^ and intensive bubbling was implemented in all rearing tanks. On D0, the day after the reproduction, nauplii were transferred into the rearing tanks at a rate of 180 per liter. Larvae were fed several times per day, according to their developmental stage. From zoea 1 to zoea 2, they were feed with microparticles (size 5–50 µm) 5 times per day and once with frozen *Tetraselmis* sp.. Larvae from zoea 3 to postlarvae, were fed twice per day, with microparticles (size 50–500 µm) and with living nauplii of *Artemia* sp. (about 20–40 per shrimp larvae per day).

### Zootechnical parameters

Twice a day (morning and afternoon), larval survival rate, larval stage and health were monitored as described in Callac et al., [24]. Briefly, the larval survival rate (LSR) was calculated by averaging 3 direct counts of the living larvae in 1 L as follow: LSR = 100 * (living larvae/ initial number of nauplii). The larval stages were established by observing 30 larvae per tank with a binocular magnifying glass as described in Callac et al., 2022 [24]. In addition, larvae health was assessed daily by observing feeding behavior, the presence of feces in the intestinal track, and visible signs of diseases [22,27]. Later in this study, we distinguished healthy from unhealthy rearings primarily based on larval survival rates compared to the established reference threshold. The reference threshold was established for a specific day using data of 10 years of successful rearing 2008–2018 made using the condition TA3 (treated water and first addition of antibiotic on day 3), without noticeable and undetermined mass larval mortality [24,25]. Rearing was considered as healthy if its survival rate was above or equal to the reference threshold, as follow: D0 = 100%, D1 between 95 and 100%, D2 ≥ 95%, D3 ≥ 90%, D4 ≥ 85%, D5 ≥ 80%, D6 ≥ 77%, D7 ≥ 75%, D8 ≥ 73% and D9 ≥ 70%. See  S1 Table for each sample health status.

### Sample collection

On D-1 (reproduction day), about 100 eggs were sampled after spawning, using sterilized pliers and stored in 2 ml sterile microtube. The day after hatching (D0), 100 nauplii were collected using a 120 µm pore size net and sterilized spoon and stored in 2 ml microtubes. Then, every morning, before the first feeding of the day, around 100 larvae were collected using the same protocol as for the nauplii. One batch of 100 individuals per tank and per day corresponds to one sample. This procedure was used for all samples irrespective of the larval health. Each sample was treated separately. Eggs and larvae were stored in 2 ml microtubes at −80°C until RNA extractions. At the end of the experiment, we had 2 eggs samples, 10 nauplii samples within which 6 qualified as healthy and 4 as unhealthy, 65 zoea samples with 24 healthy and 41 unhealthy; and 32 mysis samples with 11 healthy and 21 unhealthy.

### RNA extractions, reverse transcriptions and sequencing

RNA from the egg and whole larvae samples were extracted using the RNeasy minikit (Qiagen) according to the manufacturer’s instructions. Each sample was treated separately. Total RNAs were reverse-transcripted into complementary DNA (cDNA) using 10 µL of the total RNA (200 ng), 1 µl the reverse transcriptase M-MLV (PROMEGA) and 2 µl random hexamers at 50 µM as described in Callac et al., (2023, 2022). All cDNAs were shipped to MrDNA (Molecular Research LP, Shallowater, Texas, United States) for the PCR using the 515f-806R primers [28,29] and sequencing of the V4 hypervariable region of the 16S rRNA gene were done. Illumina HiSeq sequencing was done using a 2 x 150 bp paired-end run and with an average sequencing depth of 20k raw reads per sample. Raw sequences were demultiplexed using the fastqSplitter available on the MrDNA website (https://www.Mrdnalab.com/mrdnafreesoftware/fastq-splitter.html) [30].

### Data processing and downstream analysis

#### Sequence analysis.

The demultiplexed reads were processed using the DADA2 package [31] available in the Rstudio software. Only sequences with a quality score greater than 30 were kept. As described in Callac et al. (2023) [25], reads were filtered using several parameters: maximum excepted error (maxEE) set at 2, a maximum N (maxN) set at 0, the chimeras were removed with the consensus method and taxonomy was assigned using the Silva 138 SSU Ref NR99 database [32]. Prior to downstream analysis, sequences with no affiliation or affiliated to the *Eukaryota*, *Mitochondria* or *Chloroplasts* were removed as we wanted to assess to the active prokaryotic diversity related to the larval microbiota. Microbial analyses were conducted using Rsoftware, using R version 4.4.1 in RStudio version 2024.04.1 + 748.

#### Alpha and beta diversity.

The alpha diversity was measured using 4 indexes: Observed, Chao 1, Shannon and Inverse Simpson (InvSimpson) using the phyloseq package [33]. The Good’s coverage was calculated to estimate if the sequencing depth was sufficient (>98%). To investigate potential significant differences between larval stage and healthy status, Kruskal-Wallis tests were performed on the alpha diversity indexes, using the rstatix package. Then prior to beta diversity and further microbial analysis, the whole ASV table was normalized with the Count Per Million (CPM) method using the phyloseq package, as done in previous studies [22,24,25,30,34,35]. The beta diversity was investigated by constructing a PCoA based on a Bray-Curtis dissimilarity matrix using phyloseq packages and ggplot2 [36]. PCoA underlined clusters according to the larvae health and stage, and ANOSIM (analysis of similarity) investigated if the microbial diversities that belong to the same group of samples (healthy *versus* unhealthy, between different stages, and between different stages and status) were similar or not. ANOSIM was done using the vegan package [37]. An R-value close to “1” indicates dissimilarity between groups while R-values near “0” mean that all the compared groups are close and cannot be separated based on the microbial composition [38].

#### Venn diagrams: shared and specific microbiota; and correlation.

Prior to Venn diagrams construction, samples were divided into 2 rearing paths: healthy and unhealthy, and each encompassed 5 groups of larvae: eggs, nauplii D0 (before transfer into the rearing tanks), nauplii (after transfer into the rearing tanks), zoea and mysis. Such comparisons highlighted for each rearing, specific microbiota and a core microbiome at the ASV level. Specific and core microbiotas from each rearing, were further compared together to seek core microbiota of each larval stage. And therefore, to evidence specific ASVs that are involved in larvae health. Venn diagrams were made using the Jvenn web application tool [39] (http://bioinfo.genotoul.fr/jvenn/example.html).

Then, specific and common microbiota of the zoea and mysis as well as the core microbiota of both healthy and unhealthy larvae were used to construct a correlogram based on Spearman correlation to highlight specific taxa related to a given stage and health. Correlogram was built using the microeco R package [40].

## Results

### Larval survival rates and larval stages

Contrasted survival rates were observed among the treatments (S1 Table) except during the first days of rearing (from D0 to D3). Rearing conducted with antibiotics exhibited larval survival rates comparable to the reference [24,25], although great error bars were observed due to one tank in each triplicate exhibiting lower survival rates than the others (S1 Table). On D2, larval observations showed that all the nauplii had metamorphosed to the zoea stage. All the zoea had metamorphosed into mysis on day D7, except larvae reared with water T (treated water as explain in the Material and methods section) and without erythromycin (TSA) (S1 Table). Larvae metamorphosed into postlarvae between the afternoon of D9 and the morning of D10.

### Composition of the larval active microbiota according to the larval stage and health status

The active prokaryotic diversity associated with the larvae was investigated using HiSeq Illumina sequencing. A total of 43,986,210 sequences spans into 10,413ASVs were obtained after removal of the *Eukaryota*, *Mitochondria*, *Chloroplasts* and unassigned sequences. The Good’s coverage, with an overall average at 100% (S1 Table), revealed that the sequencing depth was sufficient.

The alpha diversity, displayed in the Fig 1 and S1 Table, showed that the ASV richness, estimated using Observed and Chao1 indexes, decreased globally as the larvae metamorphosed, while the evenness indexes, estimated with the Shannon and Inverse Simpson indexes, were similar among the conditions. To explore differences in the alpha diversity indexes between the conditions, Kruskal-Wallis tests, followed by Dunn tests when significance was observed, were performed. Only the Inverse Simpson index showed no significant differences between the treatments (p-value = 0.0724) while, Observed, Chao1 and Shannon indexes were significantly different among the conditions (p-values < 0.005), indicating that these 3 indexes were influenced by the stage and health status of the larvae. The pairwise comparisons enabled by the Dunn tests exhibited that both the egg and nauplii were significantly different from the healthy or unhealthy zoea and mysis (Table 1). The Observed, Chao1 and Shannon indexes of healthy and unhealthy zoea were also different, as the Shannon index of healthy and unhealthy mysis, indicating that the larval health status influenced the alpha diversity. No differences were observed between the eggs and the nauplii.

**Table 1:** Pairwise comparison (Dunn test) of the alpha diversity indexes according to the larval stage and health. In bold, values indicate significant differences (P < 0.05). Larvae were classified as healthy if their survival rate was above or equal to the reference threshold otherwise, they were considered as unhealthy.

Compared groups		Observed	Chao1	Shannon	InvSimpson
Egg	Nauplii	0.848	0.848	0.522	1
Egg	Healthy zoea	**0.003**	**0.003**	**0.017**	1
Egg	Unhealthy zoea	**0.034**	**0.034**	0.115	1
Egg	Healthy mysis	0.056	0.056	0.25	1
Egg	Unhealthy mysis	**0.01**	**0.01**	**0.02**	1
Nauplii	Healthy zoea	**2.00E-08**	**2.00E-08**	**0.001**	1
Nauplii	Unhealthy zoea	**4.00E-05**	**4.00E-05**	0.055	1
Nauplii	Healthy mysis	**0.001**	**0.001**	0.341	1
Nauplii	Unhealthy mysis	**7.00E-06**	**7.00E-06**	**0.001**	1
Healthy zoea	Unhealthy zoea	**0.009**	**0.009**	**0.016**	1
Healthy zoea	Healthy mysis	**0.013**	**0.013**	**0.004**	1
Healthy zoea	Unhealthy mysis	0.405	0.397	0.967	1
Unhealthy zoea	Healthy mysis	0.691	0.695	0.313	0.172
Unhealthy zoea	Unhealthy mysis	0.182	0.181	**0.039**	1
Healthy mysis	Unhealthy mysis	0.144	0.145	**0.01**	0.162

**Fig 2:**
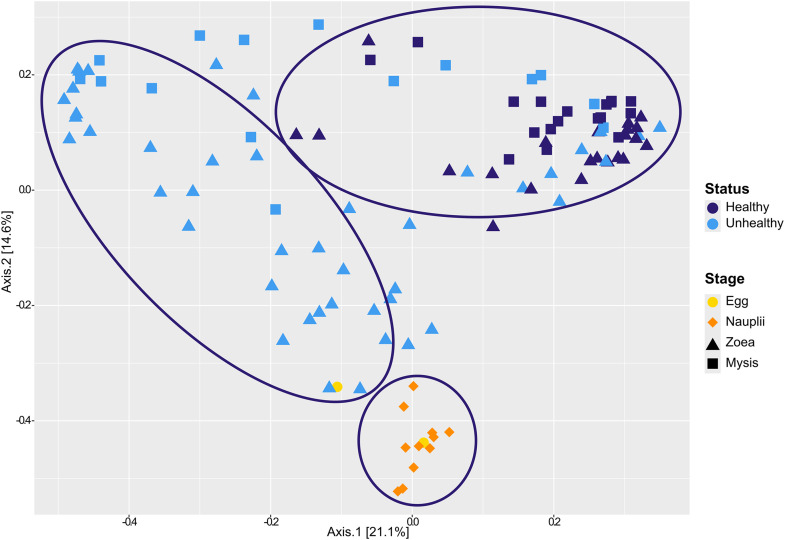
PCoA based on Bray-Curtis dissimilarity of the microbial diversity associated with the larvae. Yellow dots stand for the egg samples, orange diamonds for the nauplii, triangles for the zoea and squares for the mysis. As the mortality began at the zoea stage, the health status concerns only the zoea and the mysis, with light blue indicating healthy larvae and dark blue indicating unhealthy larvae.

The beta diversity of the whole ASV table was estimated using Bray-Curtis dissimilarity matrix and samples partitioning was visualized on PCoA (Fig 2). Three main clusters were displayed, the first one gathering all the nauplii samples and one egg sample, the second cluster encompassing both healthy zoea and healthy mysis, and the last cluster grouping all the unhealthy larvae at the zoea and mysis stage as well as several healthy larvae (Fig 2). According to the ANOSIM tests, samples clustered more by stage and health (ANOSIM R = 0.66, p-value = 0.0001) than by solely stage or health (ANOSIM R = 0.34 and 0.44 respectively, both with a p-values at 0.0001).

**Fig 3:**
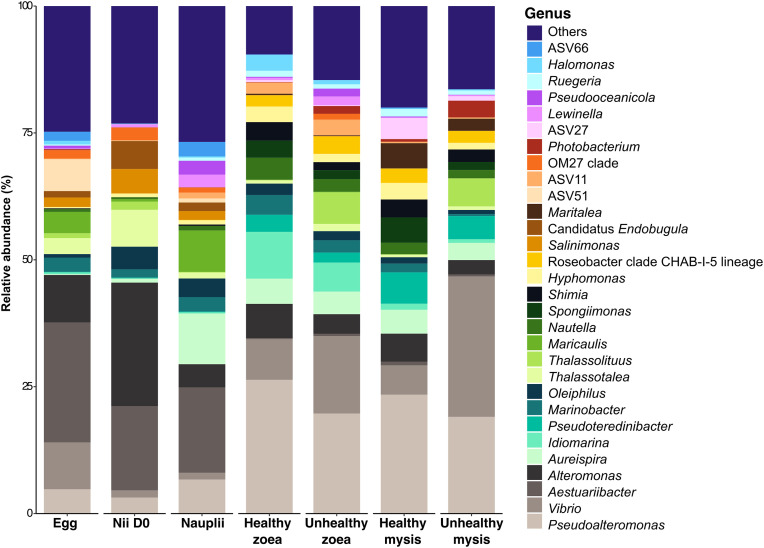
Microbial composition of the larvae during the rearing period according to the larval stage and health status. Nii D0 corresponds to the nauplii samples collected on Day 0 before transfer into the rearing tanks. Larvae were classified as healthy if their survival rate was above or equal to the reference threshold (D0 = 100%, D1 between 95 and 100%, D2 ≥ 95%, D3 ≥ 90%, D4 ≥ 85%, D5 ≥ 80%, D6 ≥ 77%, D7 ≥ 75%, D8 ≥ 73% and D9 ≥ 70%); otherwise, they were considered as unhealthy.

Among the main genera shown in Fig 3 (only the 30 most abundant genera accounting for more than 1% of the total sequence abundance are shown), the active microbiota of the eggs was mostly composed by *Aestuaribacter*, *Alteromonas*, *Vibrio* and ASV51 (*Pedosphaeraceae*). The nauplii, before their transfer into the rearing tanks, contained mainly members of *Aestuaribacter*, *Alteromonas*, *Thalassotalea, Salinimonas* and *Candidatus Endobugula* genera. Once in the rearing tanks, the nauplii’s microbiota shifted and was mainly made of bacteria from *Aestuaribacter*, *Aureispira*, *Maricualis* and *Pseudoalteromonas* genera. The microbiotas of the healthy and unhealthy larvae at the zoea and mysis stage were quite similar in terms of main genera but not regarding their relative abundance; and were different from those of the nauplii. The main taxa associated with the zoea were *Pseudoalteromonas*, *Alteromonas*, *Idiomarina*, *Thalassolituus* and *Pseudoteredinibacter,* and *Vibrio* for the mysis (Fig 3).

### Shared and specific microbiota at the ASV level according to the larval stage and health status

**Fig 4:**
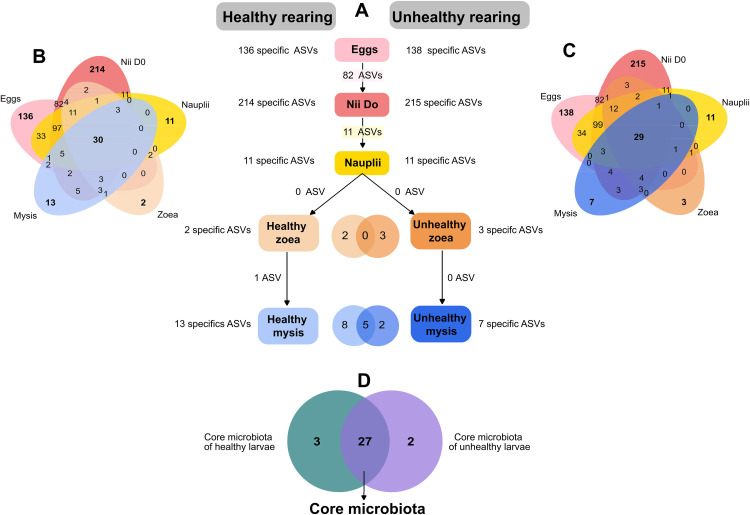
Specific and core microbiotas of the larvae according to their larval stages and health status. **(A)** Comparison of the microbiota of each larval stage and 2 consecutive larval stages between the healthy and unhealthy rearings **(B)** Venn diagram of common ASVs among all the healthy larvae samples, **(C)** Venn diagram of common ASVs among all the unhealthy larvae samples, **(D)** Venn diagram of the core microbiota of the healthy and unhealthy larvae.

To determine if vertical transmissions of bacterial lineages occurred among the larval stages, and if each larval stage displayed specific microbiotas, Venn diagrams were constructed considering the larval health and all developmental stages from the eggs to the mysis. Both healthy and unhealthy rearings (Fig 4) showed that each larval stage had its own specific microbiota with overall more specific ASVs during the early stages. Fig 4 also exhibited that 2 consecutive stages shared certain common ASVs, as well as early stages with other further developed stages, highlighting vertical transmission between the stages (see common, specific and shared ASV in S2 Table). In the same way, vertical transmission is also shown by the core microbiota indicating that these ASVs were transmitted from the early stages to the mysis. Interestingly, the egg microbiotas were almost identical between the healthy and unhealthy ones, with 2 more ASVs associated with the specific microbiota of the eggs of the unhealthy rearing (S2 Table). No common ASV was shared between unhealthy zoea and healthy mysis while one was shared between zoea and mysis in the healthy rearing. Also, no ASV was common to the healthy and unhealthy zoea. Comparison of the core microbiota of the 2 rearings revealed that they were quite similar with 3 ASVs specific of the healthy rearing and 2 of the unhealthy rearing (Fig 4 and S2 Table).

Colored ellipses are related to specific ASVs, for (A), in pink = eggs, dark pink = nauplii collected on D0, yellow = nauplii collected on D1, light orange = healthy zoea, dark orange = . Unhealthy zoea light blue = healthy mysis and dark blue = unhealthy mysis; for (B) and (C), pink = eggs, dark pink = nauplii collected on D0, yellow = nauplii collected on D1, orange = zoea, blue = mysis; and for (D), dark green = core microbiota of the healthy larvae, purple = core microbiota of the unhealthy larvae and dark purple = core microbiota common to healthy and unhealthy larvae. Numbers inside the ellipses and in the overlapping represent the number of ASVs of a given condition. Larvae were classified as healthy if their survival rate was above or equal to the reference threshold (D0 = 100%, D1 between 95 and 100%, D2 ≥ 95%, D3 ≥ 90%, D4 ≥ 85%, D5 ≥ 80%, D6 ≥ 77%, D7 ≥ 75%, D8 ≥ 73% and D9 ≥ 70%); otherwise, they were considered as unhealthy.

**Fig 5:**
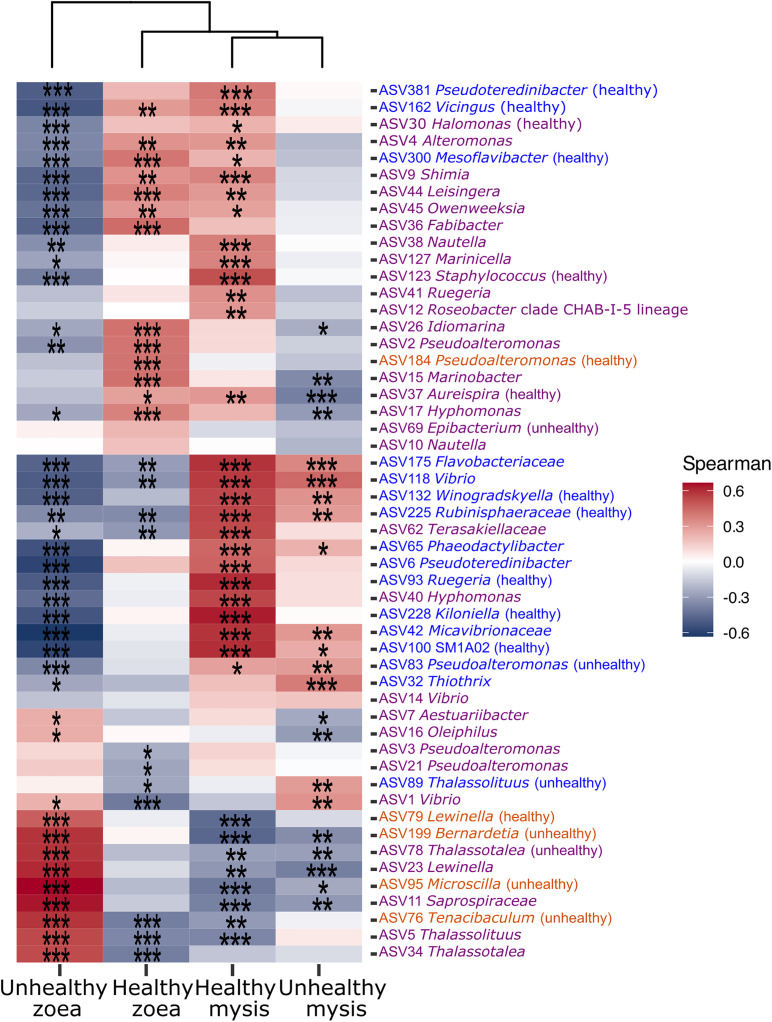
Correlogram of the specific or core microbiotas detected with the Venn diagram according to the larval stage (zoea or mysis) and health. Taxa in orange were found as specific ASVs of the zoea, taxa in blue were found as specific ASVs of the mysis and taxa in purple were part of the core microbiota. In bracket, healthy or unhealthy meant that the taxon was specific to healthy or unhealthy larvae. Larvae were classified as healthy if their survival rate was above or equal to the reference threshold (D0 = 100%, D1 between 95 and 100%, D2 ≥ 95%, D3 ≥ 90%, D4 ≥ 85%, D5 ≥ 80%, D6 ≥ 77%, D7 ≥ 75%, D8 ≥ 73% and D9 ≥ 70%); otherwise, they were considered as unhealthy. Heatmap color gradient is linked to Spearman correlation coefficient intensity: red stands for positive correlation, while blue corresponds to negative correlation. Significant correlations are noted with an asterisk (*), with no asterisk: p > 0.05, *: p ≤ 0.05, ***: p ≤ 0.001.

All lineages found to be specific to the zoea and mysis irrespective of the health status and of the core microbiota of the healthy and unhealthy rearings were used to build a correlogram (Fig 5). It showed that besides specific lineages specific of a given condition (see Venn diagram), ASVs common to all the mysis and from the core microbiota were partitioned between the healthy or unhealthy zoea and mysis. Several lineages such as ASV36 (*Fabibacter*), ASV15 (*Marinobacter*) or ASV2 (*Pseudoalteromonas*) were positively correlated with the healthy zoea, while ASV11 (*Saprospiraceae*), ASV34 (*Thalassotalea*) or ASV23 (*Lewinella*) were positively correlated with unhealthy zoea. ASV42 (*Micavibrionaceae*), ASV175 (*Flavobacteriaceae*) and ASV118 (*Vibrio*) for example were linked to healthy mysis. The unhealthy mysis were slightly positively correlated with less ASVs than the other conditions. Among the few ASVs linked to the unhealthy mysis there were ASV32 (*Thiothrix*) or ASV1 (*Vibrio*) (Fig 5).

## Discussion

*Penaeus stylirostris* shrimp production in New Caledonia has been facing important issues for the past 2 decades and it is getting difficult to provide postlarvae to the farms. Consequently, hints to understand the mortality process are of great importance. In this aim we investigated the active microbiota associated with healthy and unhealthy larvae. Indeed, RNA approach allows to study the active microbial diversity, capture the living microbiota assemblage and realize rearing surveys [22,24,25,34,41–43].

Throughout the experiment, we observed contrasted survival rates at zoea and mysis stages (S1 Table), with shifts in the main bacterial genera associated with the larvae according to their stage and health (Fig 2). According to the ANOSIM analysis, microbial partitioning was associated with larval ontogeny, health, and mainly by the combined effects of larval stage and health. Thus, our data indicated that the larval health influenced the larval microbiota with significant differences between richness indexes (Observed and Chao1) of the nauplii and of the other stages. Differences were also observed between 2 different health status of the same larvae stage (Fig 1 and S1 Table). In addition, healthy and unhealthy mysis exhibited clear statistical differences in evenness values (Shannon index) indicating that the larval microbiota was composed by a greater prokaryotic diversity in healthy larvae (higher Shannon index) than in unhealthy larvae (lower Shannon index). The same has been shown in the gut microbiota of healthy and diseased *P. vannamei* shrimps [44]. In a study focusing on *P. vannamei* larvae affected by acute hepatopancreatic necrosis disease, the authors also found statistical differences between healthy and diseased mysis with, as in our study, higher Shannon index values in healthy larvae [45]. The spread of Shannon index spectra in unhealthy zoea can be related to the upcoming mortalities during the zoea stage, which continues into the mysis stage, where mortalities become more pronounced (Fig 1). This differs from the study conducted by Zheng and colleagues or by Reyes and collaborators, where they found no differences in alpha diversity index (Observed, Chao1 and Shannon) between healthy and diseased *P. vannamei* larvae [27,45]. However, in the previously cited studies, they compared pooled larvae from different stages while we looked at each stage separately. Our data highly suggest that the larval microbiota, regardless of the developmental stage, was influenced by the larval health with an overall loss of prokaryotic richness and evenness in the unhealthy larvae and mostly in the unhealthy mysis.

To get insight into microbial partitioning and specific lineages related to the health status of the larvae according to their larval stage, comparisons were made (Fig 4 and S2 Table). When considering healthy zoea and unhealthy zoea independently, only few ASVs were specific to each condition. Indeed, 2 ASVs were specific to the healthy zoea: ASV79 affiliated to *Lewinella* and ASV184 to *Pseudoalteromonas*. *Lewinella* has been found as one of the main genera in AHPND-affected *Penaeus vannamei* larvae [46], as well as in adults affected by *V. parahaemolyticus* (non-AHPND *V. parahaemolyticus* strain) [47,48]. This suggests that this genus may play a protective role against pathogens or against opportunistic microorganisms to avoid dysbiosis. Similarly, *Pseudoalteromonas*, known to have probiotic activities [49–51] might help to maintain larval health and to promote shrimp microbiota stabilization [52,53]. Three ASVs were specific to the unhealthy zoea: *Tenacibaculum* (ASV76), *Microscilla* (ASV95) and *Bernardetia* (ASV199). *Tenacibaculum* was previously found as a biomarker of the healthy *P. stylirostris* mysis in the larvae and in the rearing water [25,35]. This genus has been detected in healthy *P. vannamei* and *P. monodon* larvae [54–56]; while it was enriched in the gut of *P. vannamei* with “cotton shrimp-like” disease [57]. *Microscilla* was positively correlated with *Acropora palmata* coral affected with white band type I disease [58]. *Bernardetia* is a marine genus found in various marine settings such as costal seawater, sea water aquarium [59], associated with sea-ice microalgae [60], in rearing tanks of Atlantic Sea scallops’ larvae (*Placopecten magellanicus*) [61], or as member of the post larvae turbot (*Scophthalmus maximus*) microbiota [62]; and was so far never associated to shrimp. These three ASVs were also positively correlated with this given status, meaning they were related to mortality and affected the zoea (Fig. 5). Thus, these three ASVs may be indicators of dysbiosis or opportunistic overgrowth during larval vulnerability, though their pathogenic potential remains to be established experimentally. Indeed, further work are needed, such as isolating and describing these species, or genome sequencing, to determine their physiological preferences, metabolism and activities, to validate their ecological functions. This will also help to establish whether they produce secondary metabolites and/or have antimicrobial activities. Indeed, *Tenacibaculum discolor* sv11 produce phenethylamine alkaloids that have antimicrobial activities against various Gram-positive bacteria (*Bacillus*, *Mycobacterium*, *Listeria*) [63], while *Tenacibaculum sp.* A4K-17 produces tenacibactins (siderophores) that are believed to play a role in *Tenacibaculum* pathogenicity towards marine farmed fishes [64]. Another hypothesis is that these 3 lineages might also directly affect the larvae by influencing the larvae homeostasis through microbial response toward a perturbation (stress, infection, tissue deterioration) and, by triggering the immune response of the larvae [65–68]. This hypothesis can be tested once these strains have been isolated by quantifying shrimp immune effector in the hemolymph after exposure to these specific taxa. In addition, no common ASV was transmitted from the unhealthy zoea to the unhealthy mysis, suggesting that the mysis death could be link to another dysbiosis process involving other lineages or could be part of an establish pathobiome. Also, the larval ontogeny might influence the larval microbiota. Indeed, larvae-microbiota interact at a given larval stage and larvae might modulate their microbiota through selection of specific lineages [27,34,35,69], along with the modulation of the host immune system [70,71]. Among the ASVs specific of the mysis, only 2 were specific of the unhealthy mysis: ASV83 related to the *Pseudoalteromonas* and ASV89 affiliated to *Thalassolituus*. As written above, *Pseudoalteromonas* seemed to be beneficial to the larvae and in this case might act as a defender against, stressors or microorganisms involved in the dysbiosis through probiotic activities. Probiotic activities could be valuable for the host’s microbiota as it is recognized that probiotics promote microbiota stabilization even when perturbed [52,53]. Indeed, *Pseudoalteromonas* species produce antimicrobial compounds such as bacteriocins that can inhibit the growth of some *Vibrio* species or Gram-positive bacteria for example; and can therefore improve or help the holobiont homeostasis [49,72,73]. However, its probiotic potential needs to be established experimentally to validate its beneficial effect on the host’s health. *Thalassolituus* also might be beneficial*.* Indeed*, Thalassolituus* have been found in the egg and nauplius microbiotas of *P. stylirostris* larvae [30] as well as in the nauplius and zoea microbiotas of *P. vannamei* [56]. According to these studies *Thalassolitus* could be beneficial for the early developmental stages of the shrimp through hydrocarbon degradation, as *Thalassolituus* are hydrocarbonoclastic microorganisms [74]. In addition, the correlogram showed that few ASVs were positively correlated with the unhealthy mysis suggesting that the dysbiosis had affected their microbiota composition and abundance.

Comparison between the core microbiota of the healthy and unhealthy rearings, indicated that few ASVs were indeed specific of the health status of the rearing (Fig 4 and S2 Table). Three ASV: ASV30 affiliated to *Halomonas* genus, ASV37 to *Aureispira* and ASV123 to *Staphylococcus* were specific to the healthy rearing. *Halomonas* sp. B12 added in the diet of *Fenneropenaeus chinensis* challenged with white spot syndrome virus showed its probiotic activity by preventing white spot syndrome virus infection [75]. This suggests that *Halomonas* might also act as probiotic in *P. stylirostris* larvae. *Aureispira,* known for its ixotrophic activity against bacteria prey such as *Vibrio* species [76,77], had previously been found in the microbiota of *P. stylirostris* healthy larvae [30,35], as well as in zoea and mysis larvae *of P. monodon* [54]. Through its ixotrophic activity, *Aureispira* might also support the stabilization of the larval microbiota. *Staphylococcus* was among the main genera in the gut microbiota of *P. vannamei* when *bacilli* and lactic acid bacteria were added in the rearing water, allowing a better survival rate compared to the control [78]. Whereas, in *P. vannamei* infected with AHPND, *Staphylococcus* was enriched in their gut microbiota [79]. That suggests that *Staphylococcus* along with *Halomonas* and *Aureispira* might defend the host against opportunistic microorganisms or pathogen to prevent dysbiosis. In the unhealthy rearing, 2 ASVs were specific: ASV69 related to *Epibacterium* not yet associated with diseased marine animals, and ASV78 related to *Thalassotalea* which was abundantly found in *P. vannamei* larvae with zoea 2 syndrome [46]. Thus, *Epibacterium* and *Thalassotalea* might both be involved in holobiont dysbiosis. Further works need to be done to establish the real role of ASV30 (*Halomonas)* ASV37 (*Aureispira)*, ASV123 (*Staphylococcus),* ASV69 (*Epibacterium)*, and ASV78 (*Thalassotalea)* on the host health and to determine if these lineages can produce secondary metabolites, have antimicrobial activities or affect the holobiont homeostasis. Besides the specific ASVs, the correlogram indicated a partitioning of the core microbiota among the healthy and unhealthy larvae, indicating that the dysbiosis did not impact the lineages of core microbiota the same way with some being positively or negatively correlated with a given condition (Fig 5).

In addition, in the unhealthy rearings, we could also hypothesize that the necrobiome of dead larvae accumulated in the rearing tanks, due to the lack of water exchange during the larval phase, which could affect or even impair the microbiota of living larvae. Indeed, animal decomposition affects the surrounding environment by releasing various components such as organic matter, organics acids, that therefore influence the diversity and activity of the surrounding microbial community [80–83]. In a previous study, we have shown that the water microbiota of rearing tanks with high mortality was different from those with lower larval mortality, suggesting that the necrobiome associated with larval decay influenced the active microbiota of the rearing water [25]; and could therefore influence the larval microbiota through horizontal transmission. On the other hand, the necrobiome might also apply a selective pressure on the shrimp microbiota. Indeed, their decomposing activities and the molecules they release from the decomposition of dead larvae can be factors that drive changes in microbial composition of both rearing water and living larvae [81,84]. Furthermore, the succession of microorganisms in the necrobiome community can influence the rearing water microbiota as well as the microbiota of living larvae [81,84]. The necrobiome can therefore participate in changing microbial diversity and on selecting specific lineages of a given larval stage. Thus, the holobiont health seemed to be driven by larval ontogeny, larval microbiota and interactions between microbiota and host immune system, along with rearing water microbiota.

As the mortality started at the zoea stage, this stage seemed critical for the dysbiosis establishment, especially with the three ASVs specific of the unhealthy zoea known to be part of the microbiome of diseased animals. It is therefore probably at this stage that dysbiosis of the microbiota could evolve into larval pathobiome. Therefore, understanding how dysbiosis of larval associated microorganisms could lead to a pathobiome and to larval death remains to be determined. Further work is required, including isolation of ASVs specific to unhealthy zoea and mysis stages, followed by experimental infections to confirm or refute their pathogenicity toward larvae at specific developmental stages. If their pathogenicity is proved, this could help to overcome larval mortality in hatcheries by guiding the development of targeted strategies, such as the use of specific probiotics, to counter these bacterial lineages.

## Supporting information

S1 Fig Schema of the water treatment used for the experiment. Schema shows the different paths with different filtration systems, with or without skimmer or bubbling to obtain Water T or Water NT used to fill the rearing tanks for which antibiotic was added for the first time on D0 (rearing tanks were filled on day 0) or on D3 of the rearing, or not. Water T corresponds to seawater that has been pumped from the lagoon the primary reservoir and then passed through a sand filter and a membrane filter of 10µm before being pulled into the secondary reservoir, then the water continuously passed through a skimmer and a series of filters: 5µm and 1µm; before being added in the rearing tanks the water circulated through a UV chamber. Water NT corresponds to the to seawater that has been pumped from the lagoon the primary reservoir and then passed through a sand filter and a membrane filter of 10µm before being pulled into the secondary reservoir, where an extensive bubbling was implemented; and before being added in the rearing tanks the water circulated through a 5µm membrane filter. Erythromycin was added at 2 ppm on D0 and then on days D3, D5, D7 and D9, or on D3 and further on days D5, D7 and D9. Five rearing water conditions were tested: 1) Water T without antibiotic named as TSA, 2) Water T with antibiotic added firstly on D3 named as TA3, 3) Water NT without antibiotic named as NTSA, 4) Water NT with antibiotic added firstly on D0 named as NTA0 and 5) Water NT with antibiotic added firstly on D3 named as NTA3.
(TIFF)

S1 Table Metadata and alpha diversity of the larval samples. Nii means nauplii collected on D0, NT means that the tanks were filled with non-treated (NT, see figure 1 for treatment used) water, T means that the tanks were filled with treated (T, see figure 1 for treatment used) water, A0 means that antibiotic was first added on D0, A3 means that antibiotic was first added on D3, SA means without antibiotics and L corresponds to larvae. Larval stage corresponds to the larval development observed during the morning monitoring. The health status of each sample was established according to the survival rate determine for the given day compared to the reference threshold: if equal or above the samples are classified as healthy if not as unhealthy. The percentage of survival for the reference threshold is as follow: D0 = 100%, D1 between 95 and 100%, D2 ≥ 95%, D3 ≥ 90%, D4 ≥ 85%, D5 ≥ 80%, D6 ≥ 77%, D7 ≥ 75%, D8 ≥ 73% and D9 ≥ 70%.
(DOCX)

S2 Table Comparison of the specific microbiota of the healthy and unhealthy series and of their core microbiota. In yellow, ASVs that were specific to the condition.
(DOCX)
