# Supplementary figures and images for: Keystone active bacterial lineages associated with Penaeus stylirostris shrimp health across larvae stages
Source: PLoS One. 2025 Oct 29;20(10):e0335417. doi: 10.1371/journal.pone.0335417 (PMC12571323; doi:10.1371/journal.pone.0335417)

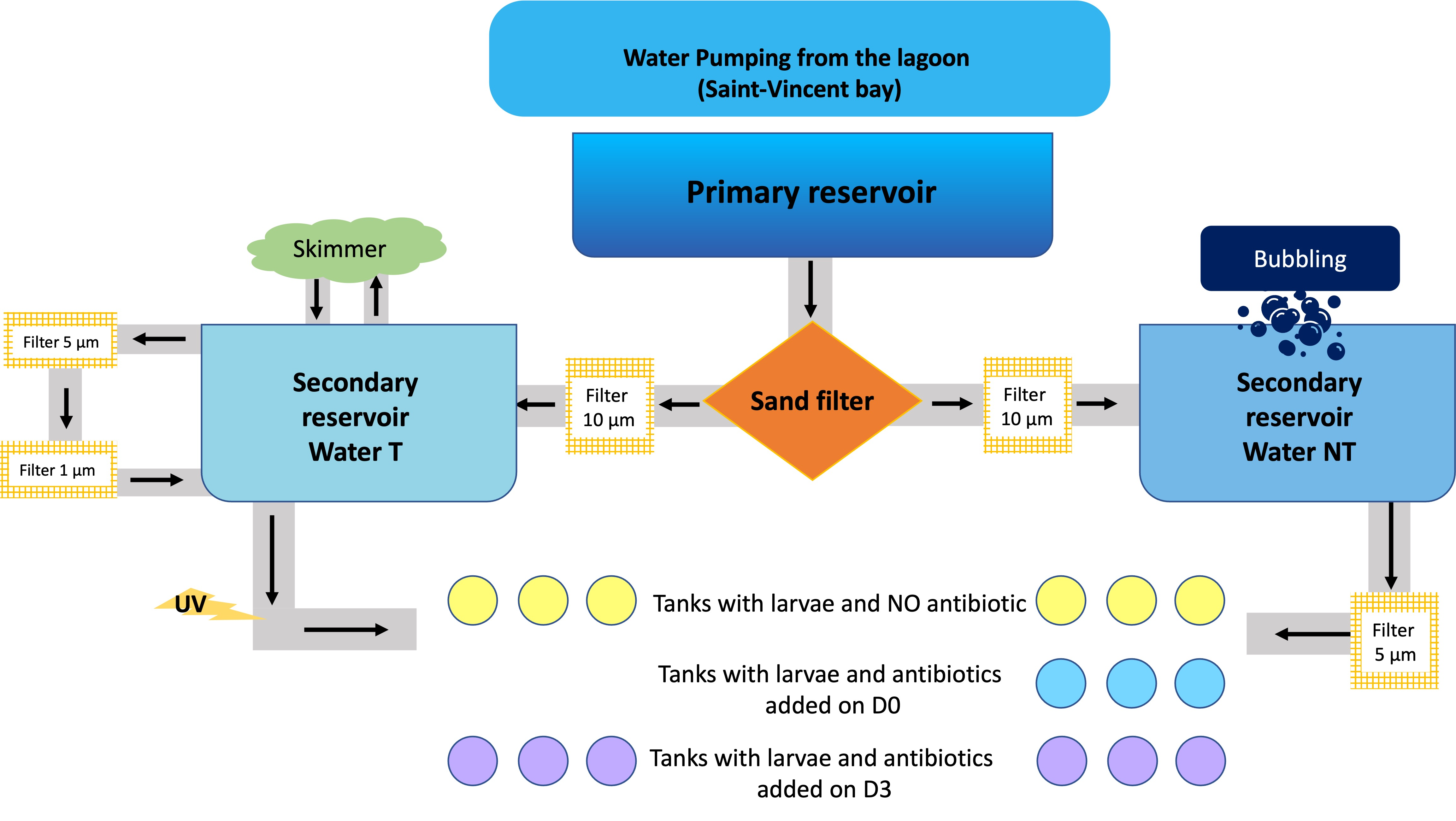

Supplement: S1 Fig — Schema shows the different paths with different filtration systems, with or without skimmer or bubbling to obtain Water T or Water NT used to fill the rearing tanks for which antibiotic was added for the first time on D0 (rearing tanks were filled on day 0) or on D3 of the rearing, or not. Water T corresponds to seawater that has been pumped from the lagoon the primary reservoir and then passed through a sand filter and a membrane filter of 10µm before being pulled into the secondary reservoir, then the water continuously passed through a skimmer and a series of filters: 5µm and 1µm; before being added in the rearing tanks the water circulated through a UV chamber. Water NT corresponds to the to seawater that has been pumped from the lagoon the primary reservoir and then passed through a sand filter and a membrane filter of 10µm before being pulled into the secondary reservoir, where an extensive bubbling was implemented; and before being added in the rearing tanks the water circulated through a 5µm membrane filter. Erythromycin was added at 2 ppm on D0 and then on days D3, D5, D7 and D9, or on D3 and further on days D5, D7 and D9. Five rearing water conditions were tested: 1) Water T without antibiotic named as TSA, 2) Water T with antibiotic added firstly on D3 named as TA3, 3) Water NT without antibiotic named as NTSA, 4) Water NT with antibiotic added firstly on D0 named as NTA0 and 5) Water NT with antibiotic added firstly on D3 named as NTA3. (TIFF) [file pone.0335417.s001.tiff]
