# Supplementary material for: Keystone active bacterial lineages associated with Penaeus stylirostris shrimp health across larvae stages
Source: PLoS One. 2025 Oct 29;20(10):e0335417. doi: 10.1371/journal.pone.0335417 (PMC12571323; doi:10.1371/journal.pone.0335417)
Supplement: S1 Table — Nii means nauplii collected on D0, NT means that the tanks were filled with non-treated (NT, see figure 1 for treatment used) water, T means that the tanks were filled with treated (T, see figure 1 for treatment used) water, A0 means that antibiotic was first added on D0, A3 means that antibiotic was first added on D3, SA means without antibiotics and L corresponds to larvae. Larval stage corresponds to the larval development observed during the morning monitoring. The health status of each sample was established according to the survival rate determine for the given day compared to the reference threshold: if equal or above the samples are classified as healthy if not as unhealthy. The percentage of survival for the reference threshold is as follow: D0 = 100%, D1 between 95 and 100%, D2 ≥ 95%, D3 ≥ 90%, D4 ≥ 85%, D5 ≥ 80%, D6 ≥ 77%, D7 ≥ 75%, D8 ≥ 73% and D9 ≥ 70%. (DOCX) [file pone.0335417.s002.docx]

| Sample | Rearing day | Survival rates (%) | Larval stages | Health status | Observed | Chao1 | Shannon | Invsimpson | Good's coverage % |
| --- | --- | --- | --- | --- | --- | --- | --- | --- | --- |
| Egg1 | D-1 | 100 | Egg | Healthy | 624 | 624 | 3.80 | 15.44 | 100 |
| Egg2 | D-1 | 100 | Egg | Healthy | 987 | 987 | 3.77 | 8.84 | 100 |
| Nii1 D0 | D0 | 100 | Nauplii | Healthy | 607 | 607 | 3.51 | 10.75 | 100 |
| Nii2 D0 | D0 | 100 | Nauplii | Healthy | 590 | 590 | 3.51 | 10.60 | 100 |
| NTA0L_D1A | D1 | 100 | Nauplii | Healthy | 720 | 720 | 3.22 | 5.96 | 100 |
| NTA0L_D1B | D1 | 92.4 | Nauplii | Unhealthy | 824 | 824 | 3.62 | 8.37 | 100 |
| NTA0L_D1C | D1 | 90.7 | Nauplii | Unhealthy | 505 | 505 | 1.89 | 2.25 | 100 |
| NTA0L_D2A | D2 | 100 | Zoea | Healthy | 288 | 288 | 2.58 | 6.67 | 100 |
| NTA0L_D2B | D2 | 89.8 | Zoea | Unhealthy* | 233 | 233 | 2.81 | 9.26 | 100 |
| NTA0L_D2C | D2 | 96.9 | Zoea | Healthy | 360 | 360.11 | 3.14 | 11.94 | 100 |
| NTA0L_D3A | D3 | 96.3 | Zoea | Healthy | 331 | 331 | 3.10 | 13.19 | 100 |
| NTA0L_D3B | D3 | 92.2 | Zoea | Healthy | 227 | 227 | 2.81 | 9.22 | 100 |
| NTA0L_D3C | D3 | 98.0 | Zoea | Healthy | 333 | 333 | 3.03 | 10.57 | 100 |
| NTA0L_D4A | D4 | 87.0 | Zoea | Healthy | 375 | 375 | 3.02 | 10.08 | 100 |
| NTA0L_D4B | D4 | 96.7 | Zoea | Healthy | 308 | 308.43 | 2.95 | 8.99 | 100 |
| NTA0L_D4C | D4 | 109.1 | Zoea | Healthy | 356 | 356 | 3.26 | 14.54 | 100 |
| NTA0L_D5A | D5 | 82.6 | Zoea | Healthy | 335 | 335 | 2.68 | 6.51 | 100 |
| NTA0L_D5B | D5 | 87.4 | Zoea | Healthy | 331 | 331 | 3.16 | 12.30 | 100 |
| NTA0L_D5C | D5 | 91.1 | Zoea | Healthy | 246 | 246 | 2.22 | 4.07 | 100 |
| NTA0L_D6A | D6 | 72.6 | Zoea | Unhealthy | 380 | 380 | 3.24 | 13.81 | 100 |
| NTA0L_D6B | D6 | 73.3 | Zoea | Unhealthy | 260 | 260 | 1.37 | 1.75 | 100 |
| NTA0L_D6C | D6 | 80.7 | Zoea | Healthy | 527 | 527 | 3.49 | 16.64 | 100 |
| NTA0L_D7A | D7 | 78.1 | Mysis | Healthy | 339 | 339 | 3.54 | 19.29 | 100 |
| NTA0L_D7B | D7 | 69.6 | Mysis | Unhealthy | 250 | 250 | 3.52 | 21.17 | 100 |
| NTA0L_D7C | D7 | 66.5 | Mysis | Unhealthy | 291 | 291 | 3.37 | 16.11 | 100 |
| NTA0L_D8A | D8 | 73.3 | Mysis | Healthy | 373 | 373 | 3.49 | 17.93 | 100 |
| NTA0L_D8B | D8 | 72.8 | Mysis | Healthy | 328 | 328 | 2.85 | 7.71 | 100 |
| NTA0L_D8C | D8 | 73.3 | Mysis | Healthy | 366 | 366.08 | 2.80 | 7.26 | 100 |
| NTA0L_D9A | D9 | 75.9 | Mysis | Healthy | 545 | 545 | 3.30 | 8.34 | 100 |
| NTA0L_D9B | D9 | 70.9 | Mysis | Healthy | 577 | 577 | 3.21 | 9.45 | 100 |
| NTA0L_D9C | D9 | 66.5 | Mysis | Unhealthy | 633 | 633.38 | 3.07 | 8.60 | 100 |
| NTA3L_D4A | D4 | 89.3 | Zoea | Healthy | 315 | 315 | 2.42 | 4.76 | 100 |
| NTA3L_D4B | D4 | 86.3 | Zoea | Healthy | 404 | 404.16 | 2.86 | 8.31 | 100 |
| NTA3L_D4C | D4 | 93.1 | Zoea | Healthy | 278 | 278 | 2.46 | 6.01 | 100 |
| NTA3L_D5A | D5 | 73.3 | Zoea | Unhealthy | 280 | 280 | 2.67 | 8.01 | 100 |
| NTA3L_D5B | D5 | 74.6 | Zoea | Unhealthy | 358 | 358 | 2.70 | 6.99 | 100 |
| NTA3L_D5C | D5 | 83.9 | Zoea | Healthy | 310 | 310 | 3.07 | 12.11 | 100 |
| NTA3L_D6A | D6 | 70.4 | Zoea | Unhealthy | 466 | 466 | 3.34 | 13.64 | 100 |
| NTA3L_D6B | D6 | 69.6 | Zoea | Unhealthy | 448 | 448 | 3.38 | 11.21 | 100 |
| NTA3L_D6C | D6 | 66.7 | Zoea | Unhealthy | 271 | 271.11 | 1.52 | 2.25 | 100 |
| NTA3L_D7A | D7 | 72.2 | Mysis | Unhealthy | 317 | 317 | 2.94 | 8.50 | 100 |
| NTA3L_D7B | D7 | 66.5 | Mysis | Unhealthy | 428 | 428 | 3.53 | 16.89 | 100 |
| NTA3L_D7C | D7 | 56.7 | Mysis | Unhealthy | 347 | 347.08 | 3.32 | 13.77 | 100 |
| NTA3L_D8A | D8 | 82.0 | Mysis | Healthy | 427 | 427 | 3.32 | 14.39 | 100 |
| NTA3L_D8B | D8 | 70.9 | Mysis | Healthy | 343 | 343 | 2.96 | 7.67 | 100 |
| NTA3L_D8C | D8 | 48.0 | Mysis | Unhealthy | 214 | 216.50 | 2.41 | 5.53 | 100 |
| NTA3L_D9A | D9 | 74.6 | Mysis | Healthy | 498 | 498 | 3.59 | 17.59 | 100 |
| NTA3L_D9B | D9 | 70.4 | Mysis | Healthy | 550 | 550 | 3.91 | 24.49 | 99.99 |
| NTA3L_D9C | D9 | 37.4 | Mysis | Unhealthy | 432 | 432.04 | 2.27 | 3 | 100 |
| NTSAL_D1A | D1 | 96.1 | Nauplii | Healthy | 851 | 864.04 | 4.24 | 19.64 | 100 |
| NTSAL_D1B | D1 | 98.7 | Nauplii | Healthy | 845 | 845 | 4.39 | 23.74 | 100 |
| NTSAL_D1C | D1 | 95.6 | Nauplii | Healthy | 1628 | 1628 | 5.34 | 57.04 | 100 |
| NTSAL_D2A | D2 | 96.1 | Zoea | Healthy | 535 | 535 | 4.08 | 24.44 | 100 |
| NTSAL_D2B | D2 | 96.9 | Zoea | Healthy | 495 | 495 | 3.94 | 25.99 | 100 |
| NTSAL_D2C | D2 | 97.4 | Zoea | Healthy | 445 | 445 | 4.17 | 31.86 | 100 |
| NTSAL_D3A | D3 | 86.9 | Zoea | Unhealthy | 485 | 485 | 3.74 | 17.20 | 100 |
| NTSAL_D3B | D3 | 86.9 | Zoea | Unhealthy | 537 | 538.13 | 3.83 | 21 | 100 |
| NTSAL_D3C | D3 | 93.7 | Zoea | Healthy | 466 | 466.07 | 3.64 | 16.55 | 100 |
| NTSAL_D4A | D4 | 65.9 | Zoea | Unhealthy | 415 | 415 | 2.75 | 5.79 | 100 |
| NTSAL_D4B | D4 | 69.6 | Zoea | Unhealthy | 373 | 373 | 2.58 | 4 | 100 |
| NTSAL_D4C | D4 | 83.9 | Zoea | Unhealthy | 532 | 532 | 3.85 | 17.65 | 100 |
| NTSAL_D5A | D5 | 40.0 | Zoea | Unhealthy | 562 | 562 | 3.63 | 10.79 | 100 |
| NTSAL_D5B | D5 | 44.3 | Zoea | Unhealthy | 465 | 465.15 | 2.50 | 3.28 | 100 |
| NTSAL_D5C | D5 | 59.8 | Zoea | Unhealthy | 506 | 506 | 2.83 | 4.34 | 100 |
| NTSAL_D6A | D6 | 27.8 | Zoea | Unhealthy | 533 | 533 | 2.41 | 2.66 | 100 |
| NTSAL_D6B | D6 | 22.8 | Zoea | Unhealthy | 436 | 436 | 2.38 | 3.37 | 100 |
| NTSAL_D6C | D6 | 38.9 | Zoea | Unhealthy | 435 | 435 | 2.12 | 2.30 | 100 |
| NTSAL_D7A | D7 | 17.2 | Mysis | Unhealthy | 436 | 436 | 2.53 | 3.73 | 100 |
| NTSAL_D7B | D7 | 12.0 | Mysis | Unhealthy | 388 | 388.07 | 3.05 | 7.66 | 100 |
| NTSAL_D7C | D7 | 21.5 | Mysis | Unhealthy | 331 | 331 | 2.01 | 2.61 | 100 |
| NTSAL_D8A | D8 | 9.8 | Mysis | Unhealthy | 285 | 285 | 1.96 | 3.44 | 100 |
| NTSAL_D8B | D8 | 5.9 | Mysis | Unhealthy | 272 | 273 | 2.02 | 3.48 | 100 |
| NTSAL_D8C | D8 | 10.4 | Mysis | Unhealthy | 437 | 437 | 2.59 | 6.04 | 100 |
| TA3L_D4A | D4 | 81.5 | Zoea | Unhealthy | 272 | 272.06 | 2.41 | 4.54 | 100 |
| TA3L_D4B | D4 | 82.0 | Zoea | Unhealthy | 287 | 287 | 2.22 | 3.78 | 100 |
| TA3L_D4C | D4 | 78.3 | Zoea | Unhealthy | 298 | 298 | 2.73 | 7.88 | 100 |
| TA3L_D5A | D5 | 68.5 | Zoea | Unhealthy | 271 | 271 | 2.32 | 4.40 | 100 |
| TA3L_D5B | D5 | 77.6 | Zoea | Unhealthy | 251 | 251 | 2.66 | 8.08 | 100 |
| TA3L_D5C | D5 | 77.6 | Zoea | Unhealthy | 248 | 248.06 | 2.57 | 6.66 | 100 |
| TA3L_D6A | D6 | 59.8 | Zoea | Unhealthy | 479 | 479 | 3.28 | 11.85 | 100 |
| TA3L_D6B | D6 | 79.4 | Zoea | Healthy | 465 | 465 | 3.24 | 14.37 | 100 |
| TA3L_D6C | D6 | 70.2 | Zoea | Unhealthy | 571 | 571.06 | 3.41 | 16.44 | 100 |
| TA3L_D7A | D7 | 61.7 | Mysis | Unhealthy | 390 | 390 | 2.92 | 7.33 | 100 |
| TA3L_D7B | D7 | 80.0 | Mysis | Healthy | 117 | 117 | 3.16 | 14.44 | 100 |
| TA3L_D7C | D7 | 62.8 | Mysis | Unhealthy | 102 | 102 | 3.27 | 18.57 | 100 |
| TA3L_D8A | D8 | 65.4 | Mysis | Unhealthy | 438 | 438 | 3.32 | 15.30 | 100 |
| TA3L_D8B | D8 | 75.7 | Mysis | Healthy | 449 | 449 | 3.23 | 14.20 | 100 |
| TA3L_D8C | D8 | 57.4 | Mysis | Unhealthy | 375 | 375 | 3.42 | 16.68 | 100 |
| TA3L_D9A | D9 | 56.1 | Mysis | Unhealthy | 663 | 663 | 3.49 | 12.65 | 100 |
| TA3L_D9B | D9 | 65.9 | Mysis | Unhealthy | 622 | 622 | 3.33 | 13.49 | 100 |
| TA3L_D9C | D9 | 57.4 | Mysis | Unhealthy | 450 | 450 | 3.28 | 12.57 | 100 |
| TSAL_D1A | D1 | 88.1 | Nauplii | Unhealthy | 531 | 531 | 2.58 | 3.79 | 100 |
| TSAL_D1B | D1 | 99.3 | Nauplii | Healthy | 631 | 631 | 4.03 | 19.04 | 100 |
| TSAL_D1C | D1 | 91.3 | Nauplii | Unhealthy | 598 | 598 | 3.77 | 13.76 | 100 |
| TSAL_D2A | D2 | 85.6 | Zoea | Unhealthy | 411 | 411 | 3.87 | 25.39 | 100 |
| TSAL_D2B | D2 | 101.7 | Zoea | Healthy | 362 | 362 | 3.78 | 25.04 | 100 |
| TSAL_D2C | D2 | 92.4 | Zoea | Healthy | 479 | 479.32 | 3.98 | 28.80 | 100 |
| TSAL_D3A | D3 | 80.0 | Zoea | Unhealthy | 380 | 380 | 3.49 | 15.42 | 100 |
| TSAL_D3B | D3 | 93.1 | Zoea | Healthy | 397 | 397 | 3.43 | 12.74 | 100 |
| TSAL_D3C | D3 | 91.1 | Zoea | Healthy | 395 | 395 | 3.79 | 23.72 | 100 |
| TSAL_D4A | D4 | 54.8 | Zoea | Unhealthy | 287 | 287 | 2.80 | 8.68 | 100 |
| TSAL_D4B | D4 | 83.9 | Zoea | Unhealthy | 352 | 352.19 | 3.19 | 12.13 | 100 |
| TSAL_D4C | D4 | 81.9 | Zoea | Unhealthy | 359 | 359 | 3.03 | 7.91 | 100 |
| TSAL_D5A | D5 | 27.0 | Zoea | Unhealthy | 435 | 435 | 3.66 | 18.89 | 100 |
| TSAL_D5B | D5 | 57.4 | Zoea | Unhealthy | 412 | 412 | 3.81 | 21.05 | 100 |
| TSAL_D5C | D5 | 53.5 | Zoea | Unhealthy | 359 | 359 | 3.32 | 10.74 | 100 |
| TSAL_D6A | D6 | 7.2 | Zoea | Unhealthy | 428 | 428 | 3.25 | 10.48 | 100 |
| TSAL_D6B | D6 | 41.3 | Zoea | Unhealthy | 419 | 419 | 3.11 | 7.47 | 100 |
| TSAL_D6C | D6 | 32.0 | Zoea | Unhealthy | 299 | 299 | 1.28 | 1.64 | 100 |
| TSAL_D7A | D7 | 3.5 | Zoea | Unhealthy | 713 | 713.14 | 4.04 | 20.84 | 100 |
| TSAL_D7B | D7 | 26.7 | Zoea | Unhealthy | 333 | 333 | 1.46 | 1.72 | 100 |
| TSAL_D7C | D7 | 18.5 | Zoea | Unhealthy | 318 | 318 | 1.07 | 1.42 | 100 |
