# Supplementary material for: Keystone active bacterial lineages associated with Penaeus stylirostris shrimp health across larvae stages
Source: PLoS One. 2025 Oct 29;20(10):e0335417. doi: 10.1371/journal.pone.0335417 (PMC12571323; doi:10.1371/journal.pone.0335417)
Supplement: S2 Table — In yellow, ASVs that were specific to the condition. (DOCX) [file pone.0335417.s003.docx]

| **Healthy** | **Unhealthy** |
| --- | --- |
| **Eggs** | **Eggs** |
| Bacteria Proteobacteria Alphaproteobacteria Rhodobacterales Rhodobacteraceae Pseudooceanicola ASV35 | Bacteria Proteobacteria Alphaproteobacteria Rhodobacterales Rhodobacteraceae Pseudooceanicola ASV35 |
| Bacteria Proteobacteria Gammaproteobacteria Enterobacterales Vibrionaceae Photobacterium ASV49 | Bacteria Proteobacteria Gammaproteobacteria Enterobacterales Vibrionaceae Photobacterium ASV49 |
| Bacteria Proteobacteria Gammaproteobacteria Enterobacterales Vibrionaceae Grimontia ASV143 | Bacteria Proteobacteria Gammaproteobacteria Pseudomonadales Moraxellaceae Acinetobacter ASV106 |
| Bacteria Proteobacteria Gammaproteobacteria Pseudomonadales Pseudomonadaceae Pseudomonas ASV182 | Bacteria Proteobacteria Gammaproteobacteria Enterobacterales Vibrionaceae Grimontia ASV143 |
| Bacteria Proteobacteria Alphaproteobacteria Rhodospirillales Terasakiellaceae ASV188 ASV188 | Bacteria Proteobacteria Gammaproteobacteria Pseudomonadales Pseudomonadaceae Pseudomonas ASV182 |
| Bacteria Proteobacteria Alphaproteobacteria Rhodobacterales Rhodobacteraceae Nioella ASV216 | Bacteria Proteobacteria Alphaproteobacteria Rhodospirillales Terasakiellaceae ASV188 ASV188 |
| Bacteria Bacteroidota Bacteroidia Chitinophagales Saprospiraceae Portibacter ASV224 | Bacteria Proteobacteria Alphaproteobacteria Rhodobacterales Rhodobacteraceae Nioella ASV216 |
| Bacteria Proteobacteria Alphaproteobacteria Rhizobiales Rhizobiaceae Allorhizobium-Neorhizobium-Pararhizobium-Rhizobium ASV265 | Bacteria Bacteroidota Bacteroidia Chitinophagales Saprospiraceae Portibacter ASV224 |
| Bacteria Proteobacteria Gammaproteobacteria Pseudomonadales Marinobacteraceae Marinobacter ASV269 | Bacteria Proteobacteria Alphaproteobacteria Rhizobiales Rhizobiaceae Allorhizobium-Neorhizobium-Pararhizobium-Rhizobium ASV265 |
| Bacteria Actinobacteriota Actinobacteria Micrococcales Micrococcaceae Kocuria ASV303 | Bacteria Proteobacteria Gammaproteobacteria Pseudomonadales Marinobacteraceae Marinobacter ASV269 |
| Bacteria Firmicutes Bacilli Lactobacillales Streptococcaceae Streptococcus ASV307 | Bacteria Actinobacteriota Actinobacteria Micrococcales Micrococcaceae Kocuria ASV303 |
| Bacteria Acidobacteriota Holophagae Acanthopleuribacterales Acanthopleuribacteraceae Acanthopleuribacter ASV316 | Bacteria Firmicutes Bacilli Lactobacillales Streptococcaceae Streptococcus ASV307 |
| Bacteria Verrucomicrobiota Chlamydiae Chlamydiales Simkaniaceae Candidatus Fritschea ASV347 | Bacteria Acidobacteriota Holophagae Acanthopleuribacterales Acanthopleuribacteraceae Acanthopleuribacter ASV316 |
| Bacteria Proteobacteria Gammaproteobacteria Pseudomonadales Halomonadaceae Chromohalobacter ASV362 | Bacteria Verrucomicrobiota Chlamydiae Chlamydiales Simkaniaceae Candidatus Fritschea ASV347 |
| Bacteria Proteobacteria Alphaproteobacteria Rhodobacterales Rhodobacteraceae ASV380 ASV380 | Bacteria Proteobacteria Gammaproteobacteria Pseudomonadales Halomonadaceae Chromohalobacter ASV362 |
| Bacteria Verrucomicrobiota Verrucomicrobiae Opitutales Puniceicoccaceae MB11C04 marine group ASV400 | Bacteria Proteobacteria Alphaproteobacteria Rhodobacterales Rhodobacteraceae ASV380 ASV380 |
| Bacteria Proteobacteria Gammaproteobacteria Salinisphaerales Salinisphaeraceae Salinisphaera ASV412 | Bacteria Actinobacteriota Actinobacteria Corynebacteriales Corynebacteriaceae Corynebacterium ASV385 |
| Bacteria Proteobacteria Alphaproteobacteria Puniceispirillales SAR116 clade ASV444 ASV444 | Bacteria Verrucomicrobiota Verrucomicrobiae Opitutales Puniceicoccaceae MB11C04 marine group ASV400 |
| Bacteria Actinobacteriota Actinobacteria Corynebacteriales Corynebacteriaceae Corynebacterium ASV478 | Bacteria Proteobacteria Gammaproteobacteria Salinisphaerales Salinisphaeraceae Salinisphaera ASV412 |
| Bacteria Bacteroidota Bacteroidia Cytophagales Flammeovirgaceae ASV501 ASV501 | Bacteria Proteobacteria Alphaproteobacteria Puniceispirillales SAR116 clade ASV444 ASV444 |
| Bacteria Proteobacteria Gammaproteobacteria Pseudomonadales SAR86 clade ASV506 ASV506 | Bacteria Actinobacteriota Actinobacteria Corynebacteriales Corynebacteriaceae Corynebacterium ASV478 |
| Bacteria Proteobacteria Gammaproteobacteria Salinisphaerales Solimonadaceae Oceanococcus ASV519 | Bacteria Bacteroidota Bacteroidia Cytophagales Flammeovirgaceae ASV501 ASV501 |
| Bacteria Firmicutes Clostridia Peptostreptococcales-Tissierellales Family XI Peptoniphilus ASV538 | Bacteria Proteobacteria Gammaproteobacteria Pseudomonadales SAR86 clade ASV506 ASV506 |
| Bacteria Proteobacteria Alphaproteobacteria Rhizobiales Stappiaceae ASV551 ASV551 | Bacteria Proteobacteria Gammaproteobacteria Salinisphaerales Solimonadaceae Oceanococcus ASV519 |
| Bacteria Actinobacteriota Actinobacteria Corynebacteriales Corynebacteriaceae Corynebacterium ASV566 | Bacteria Firmicutes Clostridia Peptostreptococcales-Tissierellales Family XI Peptoniphilus ASV538 |
| Bacteria Proteobacteria Gammaproteobacteria Enterobacterales Idiomarinaceae Idiomarina ASV574 | Bacteria Proteobacteria Alphaproteobacteria Rhizobiales Stappiaceae ASV551 ASV551 |
| Bacteria Proteobacteria Alphaproteobacteria Caulobacterales Caulobacteraceae Brevundimonas ASV585 | Bacteria Actinobacteriota Actinobacteria Corynebacteriales Corynebacteriaceae Corynebacterium ASV566 |
| Bacteria Firmicutes Bacilli Exiguobacterales Exiguobacteraceae Exiguobacterium ASV587 | Bacteria Proteobacteria Gammaproteobacteria Enterobacterales Idiomarinaceae Idiomarina ASV574 |
| Bacteria Bdellovibrionota Bdellovibrionia Bdellovibrionales Bdellovibrionaceae OM27 clade ASV593 | Bacteria Proteobacteria Alphaproteobacteria Caulobacterales Caulobacteraceae Brevundimonas ASV585 |
| Bacteria Proteobacteria Gammaproteobacteria Pseudomonadales Pseudomonadaceae Pseudomonas ASV594 | Bacteria Firmicutes Bacilli Exiguobacterales Exiguobacteraceae Exiguobacterium ASV587 |
| Bacteria Proteobacteria Alphaproteobacteria Parvibaculales PS1 clade ASV603 ASV603 | Bacteria Bdellovibrionota Bdellovibrionia Bdellovibrionales Bdellovibrionaceae OM27 clade ASV593 |
| Bacteria Firmicutes Clostridia Peptostreptococcales-Tissierellales Family XI Anaerococcus ASV637 | Bacteria Proteobacteria Gammaproteobacteria Pseudomonadales Pseudomonadaceae Pseudomonas ASV594 |
| Bacteria Actinobacteriota Actinobacteria Micrococcales Micrococcaceae Rothia ASV649 | Bacteria Proteobacteria Alphaproteobacteria Parvibaculales PS1 clade ASV603 ASV603 |
| Bacteria Planctomycetota Planctomycetes Planctomycetales Gimesiaceae ASV660 ASV660 | Bacteria Firmicutes Clostridia Peptostreptococcales-Tissierellales Family XI Anaerococcus ASV637 |
| Bacteria Proteobacteria Gammaproteobacteria Pseudomonadales Halieaceae OM60(NOR5) clade ASV661 | Bacteria Actinobacteriota Actinobacteria Micrococcales Micrococcaceae Rothia ASV649 |
| Bacteria Proteobacteria Alphaproteobacteria Caulobacterales Parvularculaceae Parvularcula ASV663 | Bacteria Planctomycetota Planctomycetes Planctomycetales Gimesiaceae ASV660 ASV660 |
| Bacteria Proteobacteria Gammaproteobacteria Pseudomonadales SAR86 clade ASV674 ASV674 | Bacteria Proteobacteria Gammaproteobacteria Pseudomonadales Halieaceae OM60(NOR5) clade ASV661 |
| Bacteria Actinobacteriota Actinobacteria Corynebacteriales Corynebacteriaceae Lawsonella ASV682 | Bacteria Proteobacteria Alphaproteobacteria Caulobacterales Parvularculaceae Parvularcula ASV663 |
| Bacteria Proteobacteria Alphaproteobacteria Micavibrionales Micavibrionaceae ASV687 ASV687 | Bacteria Proteobacteria Gammaproteobacteria Pseudomonadales SAR86 clade ASV674 ASV674 |
| Bacteria Proteobacteria Alphaproteobacteria Puniceispirillales SAR116 clade ASV695 ASV695 | Bacteria Actinobacteriota Actinobacteria Corynebacteriales Corynebacteriaceae Lawsonella ASV682 |
| Bacteria Bacteroidota Bacteroidia Chitinophagales Saprospiraceae Phaeodactylibacter ASV703 | Bacteria Proteobacteria Alphaproteobacteria Micavibrionales Micavibrionaceae ASV687 ASV687 |
| Bacteria Firmicutes Bacilli Bacillales Planococcaceae Jeotgalibacillus ASV717 | Bacteria Proteobacteria Alphaproteobacteria Puniceispirillales SAR116 clade ASV695 ASV695 |
| Bacteria Proteobacteria Gammaproteobacteria Pseudomonadales Pseudomonadaceae Pseudomonas ASV748 | Bacteria Bacteroidota Bacteroidia Chitinophagales Saprospiraceae Phaeodactylibacter ASV703 |
| Bacteria Actinobacteriota Acidimicrobiia Microtrichales Ilumatobacteraceae ASV760 ASV760 | Bacteria Firmicutes Bacilli Bacillales Planococcaceae Jeotgalibacillus ASV717 |
| Bacteria Proteobacteria Alphaproteobacteria Puniceispirillales SAR116 clade Candidatus Puniceispirillum ASV761 | Bacteria Proteobacteria Gammaproteobacteria Pseudomonadales Pseudomonadaceae Pseudomonas ASV748 |
| Bacteria Firmicutes Bacilli Bacillales Marinococcaceae Marinococcus ASV762 | Bacteria Actinobacteriota Acidimicrobiia Microtrichales Ilumatobacteraceae ASV760 ASV760 |
| Bacteria Firmicutes Bacilli Lactobacillales Aerococcaceae Aerococcus ASV776 | Bacteria Proteobacteria Alphaproteobacteria Puniceispirillales SAR116 clade Candidatus Puniceispirillum ASV761 |
| Bacteria Actinobacteriota Actinobacteria Micrococcales Micrococcaceae Rothia ASV790 | Bacteria Firmicutes Bacilli Bacillales Marinococcaceae Marinococcus ASV762 |
| Bacteria Bdellovibrionota Bdellovibrionia Bdellovibrionales Bdellovibrionaceae OM27 clade ASV793 | Bacteria Firmicutes Bacilli Lactobacillales Aerococcaceae Aerococcus ASV776 |
| Bacteria Proteobacteria Alphaproteobacteria Rhodobacterales Rhodobacteraceae Ascidiaceihabitans ASV809 | Bacteria Actinobacteriota Actinobacteria Micrococcales Micrococcaceae Rothia ASV790 |
| Bacteria Proteobacteria Alphaproteobacteria Rhizobiales Stappiaceae ASV826 ASV826 | Bacteria Bdellovibrionota Bdellovibrionia Bdellovibrionales Bdellovibrionaceae OM27 clade ASV793 |
| Bacteria Proteobacteria Gammaproteobacteria Enterobacterales Pasteurellaceae Haemophilus ASV827 | Bacteria Proteobacteria Alphaproteobacteria Rhodobacterales Rhodobacteraceae Ascidiaceihabitans ASV809 |
| Bacteria Actinobacteriota Actinobacteria Corynebacteriales Corynebacteriaceae Corynebacterium ASV857 | Bacteria Proteobacteria Alphaproteobacteria Rhizobiales Stappiaceae ASV826 ASV826 |
| Bacteria Proteobacteria Alphaproteobacteria Puniceispirillales SAR116 clade ASV873 ASV873 | Bacteria Proteobacteria Gammaproteobacteria Enterobacterales Pasteurellaceae Haemophilus ASV827 |
| Bacteria Firmicutes Clostridia Peptostreptococcales-Tissierellales Family XI Finegoldia ASV880 | Bacteria Actinobacteriota Actinobacteria Corynebacteriales Corynebacteriaceae Corynebacterium ASV857 |
| Bacteria Proteobacteria Alphaproteobacteria Azospirillales Azospirillaceae Niveispirillum ASV898 | Bacteria Proteobacteria Alphaproteobacteria Puniceispirillales SAR116 clade ASV873 ASV873 |
| Bacteria Proteobacteria Gammaproteobacteria Burkholderiales MWH-UniP1 aquatic group ASV913 ASV913 | Bacteria Firmicutes Clostridia Peptostreptococcales-Tissierellales Family XI Finegoldia ASV880 |
| Bacteria Proteobacteria Alphaproteobacteria Caulobacterales Caulobacteraceae Brevundimonas ASV914 | Bacteria Proteobacteria Alphaproteobacteria Azospirillales Azospirillaceae Niveispirillum ASV898 |
| Bacteria Proteobacteria Alphaproteobacteria Rhizobiales Hyphomicrobiaceae Filomicrobium ASV933 | Bacteria Proteobacteria Gammaproteobacteria Burkholderiales MWH-UniP1 aquatic group ASV913 ASV913 |
| Bacteria Bdellovibrionota Bdellovibrionia Bdellovibrionales Bdellovibrionaceae OM27 clade ASV942 | Bacteria Proteobacteria Alphaproteobacteria Caulobacterales Caulobacteraceae Brevundimonas ASV914 |
| Bacteria Proteobacteria Gammaproteobacteria Pseudomonadales OM182 clade ASV947 ASV947 | Bacteria Proteobacteria Alphaproteobacteria Rhizobiales Hyphomicrobiaceae Filomicrobium ASV933 |
| Bacteria Proteobacteria Gammaproteobacteria Burkholderiales Nitrosomonadaceae IS-44 ASV982 | Bacteria Bdellovibrionota Bdellovibrionia Bdellovibrionales Bdellovibrionaceae OM27 clade ASV942 |
| Bacteria Verrucomicrobiota Verrucomicrobiae Pedosphaerales Pedosphaeraceae ASV1001 ASV1001 | Bacteria Proteobacteria Gammaproteobacteria Pseudomonadales OM182 clade ASV947 ASV947 |
| Bacteria Proteobacteria Alphaproteobacteria SAR11 clade Clade I Clade Ib ASV1007 | Bacteria Proteobacteria Gammaproteobacteria Burkholderiales Nitrosomonadaceae IS-44 ASV982 |
| Bacteria Bdellovibrionota Bdellovibrionia Bacteriovoracales Bacteriovoracaceae Peredibacter ASV1020 | Bacteria Verrucomicrobiota Verrucomicrobiae Pedosphaerales Pedosphaeraceae ASV1001 ASV1001 |
| Bacteria Bacteroidota Bacteroidia Flavobacteriales Flavobacteriaceae NS4 marine group ASV1056 | Bacteria Proteobacteria Alphaproteobacteria SAR11 clade Clade I Clade Ib ASV1007 |
| Bacteria Bdellovibrionota Bdellovibrionia Bdellovibrionales Bdellovibrionaceae OM27 clade ASV1078 | Bacteria Bdellovibrionota Bdellovibrionia Bacteriovoracales Bacteriovoracaceae Peredibacter ASV1020 |
| Bacteria Firmicutes Clostridia Peptostreptococcales-Tissierellales Family XI Anaerococcus ASV1087 | Bacteria Bacteroidota Bacteroidia Flavobacteriales Flavobacteriaceae NS4 marine group ASV1056 |
| Bacteria Proteobacteria Gammaproteobacteria Pseudomonadales Pseudomonadaceae Pseudomonas ASV1089 | Bacteria Bdellovibrionota Bdellovibrionia Bdellovibrionales Bdellovibrionaceae OM27 clade ASV1078 |
| Bacteria Bdellovibrionota Bdellovibrionia Bacteriovoracales Bacteriovoracaceae Peredibacter ASV1114 | Bacteria Firmicutes Clostridia Peptostreptococcales-Tissierellales Family XI Anaerococcus ASV1087 |
| Bacteria Firmicutes Bacilli Staphylococcales Gemellaceae Gemella ASV1127 | Bacteria Proteobacteria Gammaproteobacteria Pseudomonadales Pseudomonadaceae Pseudomonas ASV1089 |
| Bacteria Actinobacteriota Actinobacteria Micrococcales Micrococcaceae Rothia ASV1141 | Bacteria Bdellovibrionota Bdellovibrionia Bacteriovoracales Bacteriovoracaceae Peredibacter ASV1114 |
| Bacteria Proteobacteria Alphaproteobacteria Thalassobaculales Thalassobaculaceae Thalassobaculum ASV1154 | Bacteria Firmicutes Bacilli Staphylococcales Gemellaceae Gemella ASV1127 |
| Bacteria Proteobacteria Alphaproteobacteria Rhodobacterales Rhodobacteraceae ASV1175 ASV1175 | Bacteria Actinobacteriota Actinobacteria Micrococcales Micrococcaceae Rothia ASV1141 |
| Bacteria Bacteroidota Bacteroidia Cytophagales Cyclobacteriaceae Ekhidna ASV1190 | Bacteria Proteobacteria Alphaproteobacteria Thalassobaculales Thalassobaculaceae Thalassobaculum ASV1154 |
| Bacteria Proteobacteria Gammaproteobacteria Xanthomonadales Xanthomonadaceae Stenotrophomonas ASV1194 | Bacteria Proteobacteria Alphaproteobacteria Rhodobacterales Rhodobacteraceae ASV1175 ASV1175 |
| Bacteria Proteobacteria Gammaproteobacteria Salinisphaerales Salinisphaeraceae Salinisphaera ASV1211 | Bacteria Bacteroidota Bacteroidia Cytophagales Cyclobacteriaceae Ekhidna ASV1190 |
| Bacteria Firmicutes Bacilli Staphylococcales Staphylococcaceae Salinicoccus ASV1242 | Bacteria Proteobacteria Gammaproteobacteria Xanthomonadales Xanthomonadaceae Stenotrophomonas ASV1194 |
| Bacteria Proteobacteria Gammaproteobacteria Pseudomonadales Marinobacteraceae Marinobacter ASV1265 | Bacteria Proteobacteria Gammaproteobacteria Salinisphaerales Salinisphaeraceae Salinisphaera ASV1211 |
| Bacteria Bdellovibrionota Bdellovibrionia Bdellovibrionales Bdellovibrionaceae OM27 clade ASV1271 | Bacteria Firmicutes Bacilli Staphylococcales Staphylococcaceae Salinicoccus ASV1242 |
| Bacteria Proteobacteria Alphaproteobacteria Caulobacterales Hyphomonadaceae Henriciella ASV1273 | Bacteria Proteobacteria Gammaproteobacteria Pseudomonadales Marinobacteraceae Marinobacter ASV1265 |
| Bacteria Proteobacteria Alphaproteobacteria Rhodospirillales Magnetospiraceae ASV1276 ASV1276 | Bacteria Bdellovibrionota Bdellovibrionia Bdellovibrionales Bdellovibrionaceae OM27 clade ASV1271 |
| Bacteria Verrucomicrobiota Verrucomicrobiae Verrucomicrobiales DEV007 ASV1316 ASV1316 | Bacteria Proteobacteria Alphaproteobacteria Caulobacterales Hyphomonadaceae Henriciella ASV1273 |
| Bacteria Firmicutes Bacilli Bacillales Planococcaceae Planococcus ASV1327 | Bacteria Proteobacteria Alphaproteobacteria Rhodospirillales Magnetospiraceae ASV1276 ASV1276 |
| Bacteria Proteobacteria Alphaproteobacteria Kiloniellales Kiloniellaceae Tistlia ASV1335 | Bacteria Verrucomicrobiota Verrucomicrobiae Verrucomicrobiales DEV007 ASV1316 ASV1316 |
| Bacteria Proteobacteria Alphaproteobacteria Tistrellales Tistrellaceae Tistrella ASV1347 | Bacteria Firmicutes Bacilli Bacillales Planococcaceae Planococcus ASV1327 |
| Bacteria Proteobacteria Alphaproteobacteria Rhodospirillales AEGEAN-169 marine group ASV1349 ASV1349 | Bacteria Proteobacteria Alphaproteobacteria Kiloniellales Kiloniellaceae Tistlia ASV1335 |
| Bacteria Proteobacteria Alphaproteobacteria Thalassobaculales Thalassobaculaceae Thalassobaculum ASV1384 | Bacteria Proteobacteria Alphaproteobacteria Tistrellales Tistrellaceae Tistrella ASV1347 |
| Bacteria Proteobacteria Gammaproteobacteria Pseudomonadales Nitrincolaceae Marinobacterium ASV1414 | Bacteria Proteobacteria Alphaproteobacteria Rhodospirillales AEGEAN-169 marine group ASV1349 ASV1349 |
| Bacteria Proteobacteria Alphaproteobacteria Rhodobacterales Rhodobacteraceae ASV1428 ASV1428 | Bacteria Proteobacteria Alphaproteobacteria Thalassobaculales Thalassobaculaceae Thalassobaculum ASV1384 |
| Bacteria Actinobacteriota Actinobacteria Corynebacteriales Corynebacteriaceae Corynebacterium ASV1486 | Bacteria Proteobacteria Gammaproteobacteria Pseudomonadales Nitrincolaceae Marinobacterium ASV1414 |
| Bacteria Proteobacteria Alphaproteobacteria Thalassobaculales Thalassobaculaceae Thalassobaculum ASV1502 | Bacteria Proteobacteria Alphaproteobacteria Rhodobacterales Rhodobacteraceae ASV1428 ASV1428 |
| Bacteria Bacteroidota Bacteroidia Flavobacteriales Flavobacteriaceae Marixanthomonas ASV1530 | Bacteria Actinobacteriota Actinobacteria Corynebacteriales Corynebacteriaceae Corynebacterium ASV1486 |
| Bacteria Cyanobacteria Cyanobacteriia Cyanobacteriales Nostocaceae Scytonema UTEX 2349 ASV1547 | Bacteria Proteobacteria Alphaproteobacteria Thalassobaculales Thalassobaculaceae Thalassobaculum ASV1502 |
| Bacteria Proteobacteria Alphaproteobacteria Thalassobaculales Nisaeaceae OM75 clade ASV1579 | Bacteria Bacteroidota Bacteroidia Flavobacteriales Flavobacteriaceae Marixanthomonas ASV1530 |
| Bacteria Proteobacteria Alphaproteobacteria Puniceispirillales SAR116 clade Candidatus Puniceispirillum ASV1580 | Bacteria Cyanobacteria Cyanobacteriia Cyanobacteriales Nostocaceae Scytonema UTEX 2349 ASV1547 |
| Bacteria Proteobacteria Alphaproteobacteria Micavibrionales Micavibrionaceae ASV1635 ASV1635 | Bacteria Proteobacteria Alphaproteobacteria Thalassobaculales Nisaeaceae OM75 clade ASV1579 |
| Bacteria Proteobacteria Alphaproteobacteria Rhizobiales Beijerinckiaceae Methylobacterium-Methylorubrum ASV1656 | Bacteria Proteobacteria Alphaproteobacteria Puniceispirillales SAR116 clade Candidatus Puniceispirillum ASV1580 |
| Bacteria Bacteroidota Bacteroidia Cytophagales Hymenobacteraceae Hymenobacter ASV1687 | Bacteria Proteobacteria Alphaproteobacteria Micavibrionales Micavibrionaceae ASV1635 ASV1635 |
| Bacteria Proteobacteria Gammaproteobacteria Burkholderiales Neisseriaceae ASV1693 ASV1693 | Bacteria Proteobacteria Alphaproteobacteria Rhizobiales Beijerinckiaceae Methylobacterium-Methylorubrum ASV1656 |
| Bacteria Proteobacteria Gammaproteobacteria Pseudomonadales Moraxellaceae Moraxella ASV1697 | Bacteria Bacteroidota Bacteroidia Cytophagales Hymenobacteraceae Hymenobacter ASV1687 |
| Bacteria Planctomycetota Planctomycetes Planctomycetales Schlesneriaceae ASV1698 ASV1698 | Bacteria Proteobacteria Gammaproteobacteria Burkholderiales Neisseriaceae ASV1693 ASV1693 |
| Bacteria Actinobacteriota Actinobacteria Corynebacteriales Corynebacteriaceae Corynebacterium ASV1701 | Bacteria Proteobacteria Gammaproteobacteria Pseudomonadales Moraxellaceae Moraxella ASV1697 |
| Archaea Crenarchaeota Nitrososphaeria Nitrosopumilales Nitrosopumilaceae Candidatus Nitrosopumilus ASV1777 | Bacteria Planctomycetota Planctomycetes Planctomycetales Schlesneriaceae ASV1698 ASV1698 |
| Bacteria Firmicutes Negativicutes Veillonellales-Selenomonadales Veillonellaceae Veillonella ASV1869 | Bacteria Actinobacteriota Actinobacteria Corynebacteriales Corynebacteriaceae Corynebacterium ASV1701 |
| Bacteria Proteobacteria Alphaproteobacteria Rhodobacterales Rhodobacteraceae ASV1991 ASV1991 | Archaea Crenarchaeota Nitrososphaeria Nitrosopumilales Nitrosopumilaceae Candidatus Nitrosopumilus ASV1777 |
| Bacteria Proteobacteria Alphaproteobacteria Rhodospirillales Magnetospiraceae ASV2027 ASV2027 | Bacteria Firmicutes Negativicutes Veillonellales-Selenomonadales Veillonellaceae Veillonella ASV1869 |
| Bacteria Proteobacteria Alphaproteobacteria Micavibrionales Micavibrionaceae ASV2056 ASV2056 | Bacteria Proteobacteria Alphaproteobacteria Rhodobacterales Rhodobacteraceae ASV1991 ASV1991 |
| Bacteria Bacteroidota Rhodothermia Balneolales Balneolaceae ASV2096 ASV2096 | Bacteria Proteobacteria Alphaproteobacteria Rhodospirillales Magnetospiraceae ASV2027 ASV2027 |
| Bacteria Proteobacteria Gammaproteobacteria Burkholderiales Neisseriaceae ASV2125 ASV2125 | Bacteria Proteobacteria Alphaproteobacteria Micavibrionales Micavibrionaceae ASV2056 ASV2056 |
| Bacteria Proteobacteria Alphaproteobacteria Rhizobiales Rhizobiaceae Aureimonas ASV2150 | Bacteria Bacteroidota Rhodothermia Balneolales Balneolaceae ASV2096 ASV2096 |
| Bacteria Bdellovibrionota Bdellovibrionia Bacteriovoracales Bacteriovoracaceae ASV2151 ASV2151 | Bacteria Proteobacteria Gammaproteobacteria Burkholderiales Neisseriaceae ASV2125 ASV2125 |
| Bacteria Bacteroidota Bacteroidia Cytophagales Hymenobacteraceae Hymenobacter ASV2156 | Bacteria Proteobacteria Alphaproteobacteria Rhizobiales Rhizobiaceae Aureimonas ASV2150 |
| Bacteria Cyanobacteria Cyanobacteriia Cyanobacteriales Nostocaceae Calothrix PCC-6303 ASV2173 | Bacteria Bdellovibrionota Bdellovibrionia Bacteriovoracales Bacteriovoracaceae ASV2151 ASV2151 |
| Bacteria Proteobacteria Gammaproteobacteria Salinisphaerales Solimonadaceae Solimonas ASV2174 | Bacteria Bacteroidota Bacteroidia Cytophagales Hymenobacteraceae Hymenobacter ASV2156 |
| Bacteria Planctomycetota Planctomycetes Pirellulales Pirellulaceae ASV2255 ASV2255 | Bacteria Cyanobacteria Cyanobacteriia Cyanobacteriales Nostocaceae Calothrix PCC-6303 ASV2173 |
| Bacteria Verrucomicrobiota Verrucomicrobiae Pedosphaerales Pedosphaeraceae SCGC AAA164-E04 ASV2408 | Bacteria Proteobacteria Gammaproteobacteria Salinisphaerales Solimonadaceae Solimonas ASV2174 |
| Bacteria Actinobacteriota Acidimicrobiia Microtrichales Microtrichaceae Sva0996 marine group ASV2465 | Bacteria Planctomycetota Planctomycetes Pirellulales Pirellulaceae ASV2255 ASV2255 |
| Bacteria Verrucomicrobiota Verrucomicrobiae Opitutales Puniceicoccaceae Verruc-01 ASV2550 | Bacteria Verrucomicrobiota Verrucomicrobiae Pedosphaerales Pedosphaeraceae SCGC AAA164-E04 ASV2408 |
| Bacteria Campylobacterota Campylobacteria Campylobacterales Campylobacteraceae Campylobacter ASV2561 | Bacteria Actinobacteriota Acidimicrobiia Microtrichales Microtrichaceae Sva0996 marine group ASV2465 |
| Bacteria Proteobacteria Gammaproteobacteria Pseudomonadales Alcanivoracaceae Ketobacter ASV2666 | Bacteria Verrucomicrobiota Verrucomicrobiae Opitutales Puniceicoccaceae Verruc-01 ASV2550 |
| Bacteria Proteobacteria Alphaproteobacteria Kiloniellales Kiloniellaceae Tistlia ASV2710 | Bacteria Campylobacterota Campylobacteria Campylobacterales Campylobacteraceae Campylobacter ASV2561 |
| Bacteria Bacteroidota Bacteroidia Chitinophagales Saprospiraceae ASV2809 ASV2809 | Bacteria Proteobacteria Gammaproteobacteria Pseudomonadales Alcanivoracaceae Ketobacter ASV2666 |
| Bacteria Verrucomicrobiota Verrucomicrobiae Opitutales Puniceicoccaceae Pelagicoccus ASV2849 | Bacteria Proteobacteria Alphaproteobacteria Kiloniellales Kiloniellaceae Tistlia ASV2710 |
| Bacteria Bacteroidota Bacteroidia Chitinophagales Saprospiraceae ASV2890 ASV2890 | Bacteria Bacteroidota Bacteroidia Chitinophagales Saprospiraceae ASV2809 ASV2809 |
| Bacteria Proteobacteria Alphaproteobacteria Rhodobacterales Rhodobacteraceae Ascidiaceihabitans ASV2969 | Bacteria Verrucomicrobiota Verrucomicrobiae Opitutales Puniceicoccaceae Pelagicoccus ASV2849 |
| Bacteria Bacteroidota Bacteroidia Cytophagales Cyclobacteriaceae Imperialibacter ASV3129 | Bacteria Bacteroidota Bacteroidia Chitinophagales Saprospiraceae ASV2890 ASV2890 |
| Bacteria Actinobacteriota Actinobacteria Corynebacteriales Dietziaceae Dietzia ASV3152 | Bacteria Proteobacteria Alphaproteobacteria Rhodobacterales Rhodobacteraceae Ascidiaceihabitans ASV2969 |
| Bacteria Proteobacteria Gammaproteobacteria Arenicellales Arenicellaceae HTCC5015 ASV3254 | Bacteria Bacteroidota Bacteroidia Cytophagales Cyclobacteriaceae Imperialibacter ASV3129 |
| Bacteria Proteobacteria Alphaproteobacteria Rhizobiales Beijerinckiaceae ASV3292 ASV3292 | Bacteria Actinobacteriota Actinobacteria Corynebacteriales Dietziaceae Dietzia ASV3152 |
| Bacteria Proteobacteria Gammaproteobacteria Salinisphaerales Solimonadaceae Fontimonas ASV3387 | Bacteria Proteobacteria Gammaproteobacteria Arenicellales Arenicellaceae HTCC5015 ASV3254 |
| Bacteria Bacteroidota Bacteroidia Sphingobacteriales Sphingobacteriaceae Pedobacter ASV3425 | Bacteria Proteobacteria Alphaproteobacteria Rhizobiales Beijerinckiaceae ASV3292 ASV3292 |
| Bacteria Proteobacteria Gammaproteobacteria Burkholderiales Burkholderiaceae Cupriavidus ASV4048 | Bacteria Proteobacteria Gammaproteobacteria Salinisphaerales Solimonadaceae Fontimonas ASV3387 |
| Bacteria Proteobacteria Gammaproteobacteria Pseudomonadales Pseudomonadaceae Pseudomonas ASV4357 | Bacteria Bacteroidota Bacteroidia Sphingobacteriales Sphingobacteriaceae Pedobacter ASV3425 |
| Bacteria Proteobacteria Alphaproteobacteria Caulobacterales Hyphomonadaceae Algimonas ASV4467 | Bacteria Proteobacteria Gammaproteobacteria Burkholderiales Burkholderiaceae Cupriavidus ASV4048 |
| Bacteria Proteobacteria Alphaproteobacteria Rhodobacterales Rhodobacteraceae Albimonas ASV6168 | Bacteria Proteobacteria Gammaproteobacteria Pseudomonadales Pseudomonadaceae Pseudomonas ASV4357 |
|  | Bacteria Proteobacteria Alphaproteobacteria Caulobacterales Hyphomonadaceae Algimonas ASV4467 |
|  | Bacteria Proteobacteria Alphaproteobacteria Rhodobacterales Rhodobacteraceae Albimonas ASV6168 |
| **Microbiota common to the eggs and nauplii collected on D0** | **Microbiota common to the eggs and nauplii collected on D0** |
| Bacteria Bacteroidota Bacteroidia Flavobacteriales Flavobacteriaceae Kordia ASV43 | Bacteria Proteobacteria Gammaproteobacteria Pseudomonadales Oceanospirillaceae Oceanospirillum ASV73 |
| Bacteria Proteobacteria Alphaproteobacteria Rickettsiales Rickettsiaceae Candidatus Cryptoprodotis ASV92 | Bacteria Proteobacteria Alphaproteobacteria Rickettsiales Rickettsiaceae Candidatus Cryptoprodotis ASV92 |
| Bacteria Proteobacteria Gammaproteobacteria Enterobacterales Vibrionaceae Vibrio ASV101 | Bacteria Proteobacteria Gammaproteobacteria Pseudomonadales Marinobacteraceae Marinobacter ASV121 |
| Bacteria Proteobacteria Gammaproteobacteria Pseudomonadales Marinobacteraceae Marinobacter ASV121 | Bacteria Bacteroidota Bacteroidia Flavobacteriales Cryomorphaceae Phaeocystidibacter ASV129 |
| Bacteria Actinobacteriota Acidimicrobiia Actinomarinales Actinomarinaceae Candidatus Actinomarina ASV148 | Bacteria Actinobacteriota Acidimicrobiia Actinomarinales Actinomarinaceae Candidatus Actinomarina ASV148 |
| Bacteria Proteobacteria Gammaproteobacteria Pseudomonadales Marinomonadaceae Marinomonas ASV160 | Bacteria Proteobacteria Gammaproteobacteria Pseudomonadales Marinomonadaceae Marinomonas ASV160 |
| Bacteria Proteobacteria Alphaproteobacteria Rhodobacterales Rhodobacteraceae Sulfitobacter ASV178 | Bacteria Proteobacteria Alphaproteobacteria Rhodobacterales Rhodobacteraceae Sulfitobacter ASV178 |
| Bacteria Proteobacteria Alphaproteobacteria Rhizobiales Stappiaceae Roseibium ASV179 | Bacteria Proteobacteria Alphaproteobacteria Rhizobiales Stappiaceae Roseibium ASV179 |
| Bacteria Verrucomicrobiota Verrucomicrobiae Verrucomicrobiales Rubritaleaceae Persicirhabdus ASV185 | Bacteria Verrucomicrobiota Verrucomicrobiae Verrucomicrobiales Rubritaleaceae Persicirhabdus ASV185 |
| Archaea Nanoarchaeota Nanoarchaeia Woesearchaeales SCGC AAA286-E23 ASV186 ASV186 | Archaea Nanoarchaeota Nanoarchaeia Woesearchaeales SCGC AAA286-E23 ASV186 ASV186 |
| Bacteria Proteobacteria Alphaproteobacteria Rhodobacterales Rhodobacteraceae ASV191 ASV191 | Bacteria Proteobacteria Alphaproteobacteria Rhodobacterales Rhodobacteraceae ASV191 ASV191 |
| Bacteria Bacteroidota Bacteroidia Flavobacteriales Crocinitomicaceae Fluviicola ASV203 | Bacteria Bacteroidota Bacteroidia Flavobacteriales Crocinitomicaceae Fluviicola ASV203 |
| Bacteria Cyanobacteria Cyanobacteriia Synechococcales Cyanobiaceae Synechococcus CC9902 ASV212 | Bacteria Cyanobacteria Cyanobacteriia Synechococcales Cyanobiaceae Synechococcus CC9902 ASV212 |
| Bacteria Proteobacteria Gammaproteobacteria Pseudomonadales SAR86 clade ASV223 ASV223 | Bacteria Proteobacteria Gammaproteobacteria Pseudomonadales SAR86 clade ASV223 ASV223 |
| Bacteria Proteobacteria Gammaproteobacteria Pseudomonadales Saccharospirillaceae Litoribrevibacter ASV229 | Bacteria Proteobacteria Gammaproteobacteria Pseudomonadales Saccharospirillaceae Litoribrevibacter ASV229 |
| Bacteria Proteobacteria Gammaproteobacteria Pseudomonadales Saccharospirillaceae Bermanella ASV254 | Bacteria Proteobacteria Gammaproteobacteria Pseudomonadales Saccharospirillaceae Bermanella ASV254 |
| Bacteria Proteobacteria Gammaproteobacteria Pseudomonadales Halieaceae OM60(NOR5) clade ASV309 | Bacteria Proteobacteria Gammaproteobacteria Pseudomonadales Halieaceae OM60(NOR5) clade ASV309 |
| Bacteria Proteobacteria Gammaproteobacteria Pseudomonadales Alcanivoracaceae1 Alcanivorax ASV311 | Bacteria Proteobacteria Gammaproteobacteria Pseudomonadales Alcanivoracaceae1 Alcanivorax ASV311 |
| Bacteria Proteobacteria Alphaproteobacteria Rhodobacterales Rhodobacteraceae ASV325 ASV325 | Bacteria Proteobacteria Alphaproteobacteria Rhodobacterales Rhodobacteraceae ASV325 ASV325 |
| Bacteria Proteobacteria Alphaproteobacteria Rhodobacterales Rhodobacteraceae Marivita ASV329 | Bacteria Proteobacteria Alphaproteobacteria Rhodobacterales Rhodobacteraceae Marivita ASV329 |
| Bacteria Proteobacteria Gammaproteobacteria Pseudomonadales SAR86 clade ASV331 ASV331 | Bacteria Proteobacteria Gammaproteobacteria Pseudomonadales SAR86 clade ASV331 ASV331 |
| Bacteria Proteobacteria Gammaproteobacteria Pseudomonadales Cellvibrionaceae Teredinibacter ASV335 | Bacteria Proteobacteria Gammaproteobacteria Pseudomonadales Cellvibrionaceae Teredinibacter ASV335 |
| Bacteria Planctomycetota Planctomycetes Pirellulales Pirellulaceae Pirellula ASV338 | Bacteria Planctomycetota Planctomycetes Pirellulales Pirellulaceae Pirellula ASV338 |
| Bacteria Bacteroidota Bacteroidia Chitinophagales Saprospiraceae ASV341 ASV341 | Bacteria Bacteroidota Bacteroidia Chitinophagales Saprospiraceae ASV341 ASV341 |
| Bacteria Proteobacteria Gammaproteobacteria Pseudomonadales Alcanivoracaceae Ketobacter ASV365 | Bacteria Proteobacteria Gammaproteobacteria Pseudomonadales Alcanivoracaceae Ketobacter ASV365 |
| Bacteria Proteobacteria Alphaproteobacteria Rhodobacterales Rhodobacteraceae Marivita ASV374 | Bacteria Proteobacteria Alphaproteobacteria Rhodobacterales Rhodobacteraceae Marivita ASV374 |
| Archaea Nanoarchaeota Nanoarchaeia Woesearchaeales SCGC AAA286-E23 ASV384 ASV384 | Archaea Nanoarchaeota Nanoarchaeia Woesearchaeales SCGC AAA286-E23 ASV384 ASV384 |
| Bacteria Proteobacteria Alphaproteobacteria Micavibrionales Micavibrionaceae ASV387 ASV387 | Bacteria Proteobacteria Alphaproteobacteria Micavibrionales Micavibrionaceae ASV387 ASV387 |
| Bacteria Actinobacteriota Actinobacteria Corynebacteriales Corynebacteriaceae Corynebacterium ASV392 | Bacteria Actinobacteriota Actinobacteria Corynebacteriales Corynebacteriaceae Corynebacterium ASV392 |
| Bacteria Bdellovibrionota Bdellovibrionia Bdellovibrionales Bdellovibrionaceae OM27 clade ASV414 | Bacteria Bdellovibrionota Bdellovibrionia Bdellovibrionales Bdellovibrionaceae OM27 clade ASV414 |
| Bacteria Bacteroidota Bacteroidia Flavobacteriales Flavobacteriaceae Tenacibaculum ASV416 | Bacteria Bacteroidota Bacteroidia Flavobacteriales Flavobacteriaceae Tenacibaculum ASV416 |
| Bacteria Proteobacteria Alphaproteobacteria Rhodobacterales Rhodobacteraceae Planktotalea ASV420 | Bacteria Proteobacteria Alphaproteobacteria Rhodobacterales Rhodobacteraceae Planktotalea ASV420 |
| Bacteria Bacteroidota Bacteroidia Flavobacteriales Cryomorphaceae ASV425 ASV425 | Bacteria Bacteroidota Bacteroidia Flavobacteriales Cryomorphaceae ASV425 ASV425 |
| Bacteria Proteobacteria Alphaproteobacteria Rhodobacterales Rhodobacteraceae Sulfitobacter ASV447 | Bacteria Proteobacteria Alphaproteobacteria Rhodobacterales Rhodobacteraceae Sulfitobacter ASV447 |
| Bacteria Proteobacteria Alphaproteobacteria Caulobacterales Caulobacteraceae Caulobacter ASV449 | Bacteria Proteobacteria Alphaproteobacteria Caulobacterales Caulobacteraceae Caulobacter ASV449 |
| Bacteria Proteobacteria Gammaproteobacteria Pseudomonadales Saccharospirillaceae Oleibacter ASV484 | Bacteria Proteobacteria Gammaproteobacteria Pseudomonadales Saccharospirillaceae Oleibacter ASV484 |
| Bacteria Actinobacteriota Actinobacteria Micrococcales Microbacteriaceae Candidatus Aquiluna ASV497 | Bacteria Actinobacteriota Actinobacteria Micrococcales Microbacteriaceae Candidatus Aquiluna ASV497 |
| Bacteria Fusobacteriota Fusobacteriia Fusobacteriales Leptotrichiaceae Hypnocyclicus ASV559 | Bacteria Fusobacteriota Fusobacteriia Fusobacteriales Leptotrichiaceae Hypnocyclicus ASV559 |
| Bacteria Bdellovibrionota Bdellovibrionia Bdellovibrionales Bdellovibrionaceae OM27 clade ASV563 | Bacteria Bdellovibrionota Bdellovibrionia Bdellovibrionales Bdellovibrionaceae OM27 clade ASV563 |
| Bacteria Proteobacteria Gammaproteobacteria Pseudomonadales Porticoccaceae Porticoccus ASV569 | Bacteria Proteobacteria Gammaproteobacteria Pseudomonadales Porticoccaceae Porticoccus ASV569 |
| Bacteria Proteobacteria Gammaproteobacteria Pseudomonadales Marinobacteraceae Marinobacter ASV573 | Bacteria Proteobacteria Gammaproteobacteria Pseudomonadales Marinobacteraceae Marinobacter ASV573 |
| Bacteria Proteobacteria Gammaproteobacteria Enterobacterales Pseudoalteromonadaceae Psychrosphaera ASV602 | Bacteria Proteobacteria Gammaproteobacteria Enterobacterales Pseudoalteromonadaceae Psychrosphaera ASV602 |
| Bacteria Verrucomicrobiota Verrucomicrobiae Pedosphaerales Pedosphaeraceae SCGC AAA164-E04 ASV614 | Bacteria Verrucomicrobiota Verrucomicrobiae Pedosphaerales Pedosphaeraceae SCGC AAA164-E04 ASV614 |
| Bacteria Bdellovibrionota Bdellovibrionia Bdellovibrionales Bdellovibrionaceae OM27 clade ASV621 | Bacteria Bdellovibrionota Bdellovibrionia Bdellovibrionales Bdellovibrionaceae OM27 clade ASV621 |
| Bacteria Proteobacteria Gammaproteobacteria Pseudomonadales Saccharospirillaceae Oleispira ASV623 | Bacteria Proteobacteria Gammaproteobacteria Pseudomonadales Saccharospirillaceae Oleispira ASV623 |
| Bacteria Bacteroidota Bacteroidia Flavobacteriales Flavobacteriaceae Aquimarina ASV641 | Bacteria Bacteroidota Bacteroidia Flavobacteriales Flavobacteriaceae Aquimarina ASV641 |
| Bacteria Proteobacteria Gammaproteobacteria Methylococcales Cycloclasticaceae Cycloclasticus ASV683 | Bacteria Proteobacteria Gammaproteobacteria Methylococcales Cycloclasticaceae Cycloclasticus ASV683 |
| Bacteria Bdellovibrionota Bdellovibrionia Bdellovibrionales Bdellovibrionaceae OM27 clade ASV697 | Bacteria Bdellovibrionota Bdellovibrionia Bdellovibrionales Bdellovibrionaceae OM27 clade ASV697 |
| Bacteria Verrucomicrobiota Verrucomicrobiae Verrucomicrobiales Rubritaleaceae Roseibacillus ASV710 | Bacteria Verrucomicrobiota Verrucomicrobiae Verrucomicrobiales Rubritaleaceae Roseibacillus ASV710 |
| Bacteria Bacteroidota Bacteroidia Sphingobacteriales NS11-12 marine group ASV733 ASV733 | Bacteria Bacteroidota Bacteroidia Sphingobacteriales NS11-12 marine group ASV733 ASV733 |
| Bacteria Planctomycetota Phycisphaerae Phycisphaerales Phycisphaeraceae ASV752 ASV752 | Bacteria Planctomycetota Phycisphaerae Phycisphaerales Phycisphaeraceae ASV752 ASV752 |
| Bacteria Proteobacteria Gammaproteobacteria Pseudomonadales Hahellaceae Hahella ASV773 | Bacteria Proteobacteria Gammaproteobacteria Pseudomonadales Hahellaceae Hahella ASV773 |
| Archaea Nanoarchaeota Nanoarchaeia Woesearchaeales SCGC AAA286-E23 ASV789 ASV789 | Archaea Nanoarchaeota Nanoarchaeia Woesearchaeales SCGC AAA286-E23 ASV789 ASV789 |
| Bacteria Planctomycetota Planctomycetes Planctomycetales Gimesiaceae ASV802 ASV802 | Bacteria Planctomycetota Planctomycetes Planctomycetales Gimesiaceae ASV802 ASV802 |
| Bacteria Bacteroidota Bacteroidia Chitinophagales Saprospiraceae ASV840 ASV840 | Bacteria Bacteroidota Bacteroidia Chitinophagales Saprospiraceae ASV840 ASV840 |
| Bacteria Bacteroidota Bacteroidia Flavobacteriales Flavobacteriaceae NS3a marine group ASV902 | Bacteria Bacteroidota Bacteroidia Flavobacteriales Flavobacteriaceae NS3a marine group ASV902 |
| Bacteria Bdellovibrionota Bdellovibrionia Bacteriovoracales Bacteriovoracaceae Peredibacter ASV920 | Bacteria Bdellovibrionota Bdellovibrionia Bacteriovoracales Bacteriovoracaceae Peredibacter ASV920 |
| Bacteria Proteobacteria Gammaproteobacteria Pseudomonadales Oleiphilaceae Oleiphilus ASV923 | Bacteria Proteobacteria Gammaproteobacteria Pseudomonadales Oleiphilaceae Oleiphilus ASV923 |
| Bacteria Proteobacteria Gammaproteobacteria Enterobacterales Pseudoalteromonadaceae Algicola ASV951 | Bacteria Proteobacteria Gammaproteobacteria Enterobacterales Pseudoalteromonadaceae Algicola ASV951 |
| Bacteria Bdellovibrionota Bdellovibrionia Bdellovibrionales Bdellovibrionaceae OM27 clade ASV971 | Bacteria Bdellovibrionota Bdellovibrionia Bdellovibrionales Bdellovibrionaceae OM27 clade ASV971 |
| Bacteria Bdellovibrionota Bdellovibrionia Bdellovibrionales Bdellovibrionaceae OM27 clade ASV1006 | Bacteria Bdellovibrionota Bdellovibrionia Bdellovibrionales Bdellovibrionaceae OM27 clade ASV1006 |
| Bacteria Proteobacteria Gammaproteobacteria Pseudomonadales Cellvibrionaceae Candidatus Endobugula ASV1010 | Bacteria Proteobacteria Gammaproteobacteria Pseudomonadales Cellvibrionaceae Candidatus Endobugula ASV1010 |
| Bacteria Proteobacteria Gammaproteobacteria Enterobacterales Pseudoalteromonadaceae Algicola ASV1046 | Bacteria Proteobacteria Gammaproteobacteria Enterobacterales Pseudoalteromonadaceae Algicola ASV1046 |
| Archaea Nanoarchaeota Nanoarchaeia Woesearchaeales SCGC AAA286-E23 ASV1067 ASV1067 | Archaea Nanoarchaeota Nanoarchaeia Woesearchaeales SCGC AAA286-E23 ASV1067 ASV1067 |
| Bacteria Fusobacteriota Fusobacteriia Fusobacteriales Fusobacteriaceae Propionigenium ASV1074 | Bacteria Fusobacteriota Fusobacteriia Fusobacteriales Fusobacteriaceae Propionigenium ASV1074 |
| Archaea Nanoarchaeota Nanoarchaeia Woesearchaeales SCGC AAA286-E23 ASV1083 ASV1083 | Archaea Nanoarchaeota Nanoarchaeia Woesearchaeales SCGC AAA286-E23 ASV1083 ASV1083 |
| Bacteria Proteobacteria Alphaproteobacteria Sphingomonadales Sphingomonadaceae Erythrobacter ASV1126 | Bacteria Proteobacteria Alphaproteobacteria Sphingomonadales Sphingomonadaceae Erythrobacter ASV1126 |
| Bacteria Planctomycetota Planctomycetes Planctomycetales Gimesiaceae ASV1139 ASV1139 | Bacteria Planctomycetota Planctomycetes Planctomycetales Gimesiaceae ASV1139 ASV1139 |
| Bacteria Bdellovibrionota Bdellovibrionia Bdellovibrionales Bdellovibrionaceae OM27 clade ASV1153 | Bacteria Bdellovibrionota Bdellovibrionia Bdellovibrionales Bdellovibrionaceae OM27 clade ASV1153 |
| Bacteria Bdellovibrionota Bdellovibrionia Bdellovibrionales Bdellovibrionaceae OM27 clade ASV1162 | Bacteria Bdellovibrionota Bdellovibrionia Bdellovibrionales Bdellovibrionaceae OM27 clade ASV1162 |
| Bacteria Bdellovibrionota Bdellovibrionia Bdellovibrionales Bdellovibrionaceae OM27 clade ASV1302 | Bacteria Bdellovibrionota Bdellovibrionia Bdellovibrionales Bdellovibrionaceae OM27 clade ASV1302 |
| Bacteria Proteobacteria Alphaproteobacteria Caulobacterales Hyphomonadaceae ASV1355 ASV1355 | Bacteria Proteobacteria Alphaproteobacteria Caulobacterales Hyphomonadaceae ASV1355 ASV1355 |
| Bacteria Acidobacteriota Holophagae Acanthopleuribacterales Acanthopleuribacteraceae Acanthopleuribacter ASV1491 | Bacteria Acidobacteriota Holophagae Acanthopleuribacterales Acanthopleuribacteraceae Acanthopleuribacter ASV1491 |
| Bacteria Bdellovibrionota Bdellovibrionia Bdellovibrionales Bdellovibrionaceae OM27 clade ASV1524 | Bacteria Bdellovibrionota Bdellovibrionia Bdellovibrionales Bdellovibrionaceae OM27 clade ASV1524 |
| Bacteria Bdellovibrionota Bdellovibrionia Bdellovibrionales Bdellovibrionaceae OM27 clade ASV1528 | Bacteria Bdellovibrionota Bdellovibrionia Bdellovibrionales Bdellovibrionaceae OM27 clade ASV1528 |
| Bacteria Bdellovibrionota Bdellovibrionia Bdellovibrionales Bdellovibrionaceae OM27 clade ASV1606 | Bacteria Bdellovibrionota Bdellovibrionia Bdellovibrionales Bdellovibrionaceae OM27 clade ASV1606 |
| Bacteria Verrucomicrobiota Verrucomicrobiae Opitutales Puniceicoccaceae Verruc-01 ASV1663 | Bacteria Verrucomicrobiota Verrucomicrobiae Opitutales Puniceicoccaceae Verruc-01 ASV1663 |
| Bacteria Bdellovibrionota Bdellovibrionia Bdellovibrionales Bdellovibrionaceae OM27 clade ASV1699 | Bacteria Bdellovibrionota Bdellovibrionia Bdellovibrionales Bdellovibrionaceae OM27 clade ASV1699 |
| Bacteria Bacteroidota Bacteroidia Chitinophagales Saprospiraceae ASV1787 ASV1787 | Bacteria Bacteroidota Bacteroidia Chitinophagales Saprospiraceae ASV1787 ASV1787 |
| Bacteria Proteobacteria Alphaproteobacteria Caulobacterales Hyphomonadaceae Algimonas ASV1867 | Bacteria Proteobacteria Alphaproteobacteria Caulobacterales Hyphomonadaceae Algimonas ASV1867 |
| Bacteria Bacteroidota Bacteroidia Flavobacteriales Flavobacteriaceae Gilvibacter ASV1993 | Bacteria Bacteroidota Bacteroidia Flavobacteriales Flavobacteriaceae Gilvibacter ASV1993 |
| Bacteria Proteobacteria Alphaproteobacteria Caulobacterales Hyphomonadaceae ASV2036 ASV2036 | Bacteria Proteobacteria Alphaproteobacteria Caulobacterales Hyphomonadaceae ASV2036 ASV2036 |
| **Nauplii collectd on D0** | **Nauplii collectd on D0** |
| Bacteria Proteobacteria Gammaproteobacteria Enterobacterales Pseudoalteromonadaceae Pseudoalteromonas ASV18 | Bacteria Bacteroidota Bacteroidia Chitinophagales Saprospiraceae Aureispira ASV29 |
| Bacteria Bacteroidota Bacteroidia Chitinophagales Saprospiraceae Aureispira ASV29 | Bacteria Bdellovibrionota Bdellovibrionia Bdellovibrionales Bdellovibrionaceae OM27 clade ASV57 |
| Bacteria Bdellovibrionota Bdellovibrionia Bdellovibrionales Bdellovibrionaceae OM27 clade ASV57 | Bacteria Bacteroidota Bacteroidia Sphingobacteriales NS11-12 marine group ASV58 ASV58 |
| Bacteria Bacteroidota Bacteroidia Sphingobacteriales NS11-12 marine group ASV58 ASV58 | Bacteria Proteobacteria Alphaproteobacteria Rhodobacterales Rhodobacteraceae ASV82 ASV82 |
| Bacteria Proteobacteria Alphaproteobacteria Rhodobacterales Rhodobacteraceae ASV82 ASV82 | Bacteria Bacteroidota Bacteroidia Chitinophagales Saprospiraceae ASV84 ASV84 |
| Bacteria Bacteroidota Bacteroidia Chitinophagales Saprospiraceae ASV84 ASV84 | Bacteria Proteobacteria Alphaproteobacteria Caulobacterales Hyphomonadaceae ASV86 ASV86 |
| Bacteria Proteobacteria Alphaproteobacteria Caulobacterales Hyphomonadaceae ASV86 ASV86 | Bacteria Proteobacteria Alphaproteobacteria Rhodospirillales Terasakiellaceae ASV87 ASV87 |
| Bacteria Proteobacteria Alphaproteobacteria Rhodospirillales Terasakiellaceae ASV87 ASV87 | Bacteria Bacteroidota Bacteroidia Flavobacteriales Crocinitomicaceae Fluviicola ASV103 |
| Bacteria Planctomycetota Phycisphaerae Phycisphaerales Phycisphaeraceae SM1A02 ASV112 | Bacteria Planctomycetota Phycisphaerae Phycisphaerales Phycisphaeraceae SM1A02 ASV112 |
| Bacteria Bacteroidota Bacteroidia Flavobacteriales Flavobacteriaceae Aquibacter ASV145 | Bacteria Bacteroidota Bacteroidia Flavobacteriales Flavobacteriaceae Aquibacter ASV145 |
| Bacteria Proteobacteria Gammaproteobacteria Pseudomonadales Saccharospirillaceae Thalassolituus ASV150 | Bacteria Proteobacteria Gammaproteobacteria Pseudomonadales Saccharospirillaceae Thalassolituus ASV150 |
| Bacteria Bacteroidota Bacteroidia Flavobacteriales NS9 marine group ASV166 ASV166 | Bacteria Bacteroidota Bacteroidia Flavobacteriales NS9 marine group ASV166 ASV166 |
| Bacteria Bacteroidota Bacteroidia Chitinophagales Saprospiraceae ASV170 ASV170 | Bacteria Proteobacteria Gammaproteobacteria Enterobacterales Alteromonadaceae ASV180 ASV180 |
| Bacteria Proteobacteria Gammaproteobacteria Legionellales Legionellaceae ASV176 ASV176 | Bacteria Proteobacteria Gammaproteobacteria Pseudomonadales Halieaceae OM60(NOR5) clade ASV181 |
| Bacteria Proteobacteria Gammaproteobacteria Enterobacterales Alteromonadaceae ASV180 ASV180 | Bacteria Proteobacteria Alphaproteobacteria Sneathiellales Sneathiellaceae Sneathiella ASV192 |
| Bacteria Proteobacteria Gammaproteobacteria Pseudomonadales Cellvibrionaceae Aestuariicella ASV207 | Bacteria Proteobacteria Gammaproteobacteria Pseudomonadales Cellvibrionaceae Aestuariicella ASV207 |
| Bacteria Planctomycetota Phycisphaerae Phycisphaerales Phycisphaeraceae Phycisphaera ASV213 | Bacteria Planctomycetota Phycisphaerae Phycisphaerales Phycisphaeraceae Phycisphaera ASV213 |
| Bacteria Bacteroidota Bacteroidia Cytophagales Flammeovirgaceae ASV217 ASV217 | Bacteria Bacteroidota Bacteroidia Cytophagales Flammeovirgaceae ASV217 ASV217 |
| Bacteria Proteobacteria Gammaproteobacteria Pseudomonadales Oceanospirillaceae Oceanospirillum ASV226 | Bacteria Proteobacteria Gammaproteobacteria Pseudomonadales Oceanospirillaceae Oceanospirillum ASV226 |
| Bacteria Proteobacteria Gammaproteobacteria Gammaproteobacteria Incertae Sedis Unknown Family Candidatus Berkiella ASV236 | Bacteria Proteobacteria Gammaproteobacteria Gammaproteobacteria Incertae Sedis Unknown Family Candidatus Berkiella ASV236 |
| Bacteria Bacteroidota Bacteroidia Flavobacteriales Cryomorphaceae Vicingus ASV245 | Bacteria Proteobacteria Alphaproteobacteria Caulobacterales Parvularculaceae Parvularcula ASV244 |
| Bacteria Proteobacteria Gammaproteobacteria Pseudomonadales Saccharospirillaceae Thalassolituus ASV257 | Bacteria Bacteroidota Bacteroidia Flavobacteriales Cryomorphaceae Vicingus ASV245 |
| Bacteria Proteobacteria Gammaproteobacteria Pseudomonadales Oleiphilaceae Oleiphilus ASV259 | Bacteria Proteobacteria Gammaproteobacteria Pseudomonadales Saccharospirillaceae Thalassolituus ASV257 |
| Bacteria Proteobacteria Alphaproteobacteria Caulobacterales Hyphomonadaceae ASV264 ASV264 | Bacteria Proteobacteria Gammaproteobacteria Pseudomonadales Oleiphilaceae Oleiphilus ASV259 |
| Bacteria Bacteroidota Bacteroidia Flavobacteriales Cryomorphaceae Owenweeksia ASV268 | Bacteria Proteobacteria Alphaproteobacteria Caulobacterales Hyphomonadaceae ASV264 ASV264 |
| Bacteria Proteobacteria Gammaproteobacteria Pseudomonadales Oleiphilaceae Oleiphilus ASV273 | Bacteria Bacteroidota Bacteroidia Flavobacteriales Cryomorphaceae Owenweeksia ASV268 |
| Bacteria Proteobacteria Gammaproteobacteria Enterobacterales Vibrionaceae Vibrio ASV278 | Bacteria Proteobacteria Gammaproteobacteria Pseudomonadales Oleiphilaceae Oleiphilus ASV273 |
| Bacteria Bacteroidota Bacteroidia Chitinophagales Saprospiraceae ASV283 ASV283 | Bacteria Proteobacteria Gammaproteobacteria Enterobacterales Vibrionaceae Vibrio ASV278 |
| Bacteria Proteobacteria Gammaproteobacteria Pseudomonadales Saccharospirillaceae Thalassolituus ASV290 | Bacteria Bacteroidota Bacteroidia Chitinophagales Saprospiraceae ASV283 ASV283 |
| Bacteria Proteobacteria Gammaproteobacteria Enterobacterales Alteromonadaceae ASV292 ASV292 | Bacteria Proteobacteria Gammaproteobacteria Pseudomonadales Saccharospirillaceae Thalassolituus ASV290 |
| Bacteria Bacteroidota Bacteroidia Cytophagales Cyclobacteriaceae Fulvivirga ASV294 | Bacteria Proteobacteria Gammaproteobacteria Enterobacterales Alteromonadaceae ASV292 ASV292 |
| Bacteria Proteobacteria Gammaproteobacteria Pseudomonadales Spongiibacteraceae BD1-7 clade ASV318 | Bacteria Bacteroidota Bacteroidia Cytophagales Cyclobacteriaceae Fulvivirga ASV294 |
| Bacteria Bdellovibrionota Bdellovibrionia Bdellovibrionales Bdellovibrionaceae OM27 clade ASV319 | Bacteria Proteobacteria Gammaproteobacteria Pseudomonadales Spongiibacteraceae BD1-7 clade ASV318 |
| Bacteria Proteobacteria Alphaproteobacteria Rhodobacterales Rhodobacteraceae ASV320 ASV320 | Bacteria Bdellovibrionota Bdellovibrionia Bdellovibrionales Bdellovibrionaceae OM27 clade ASV319 |
| Bacteria Bacteroidota Bacteroidia Chitinophagales Saprospiraceae Lewinella ASV324 | Bacteria Proteobacteria Alphaproteobacteria Rhodobacterales Rhodobacteraceae ASV320 ASV320 |
| Bacteria Proteobacteria Gammaproteobacteria Pseudomonadales MBAE14 ASV334 ASV334 | Bacteria Bacteroidota Bacteroidia Chitinophagales Saprospiraceae Lewinella ASV324 |
| Bacteria Proteobacteria Gammaproteobacteria Enterobacterales Colwelliaceae Thalassotalea ASV349 | Bacteria Proteobacteria Gammaproteobacteria Pseudomonadales MBAE14 ASV334 ASV334 |
| Bacteria Bdellovibrionota Bdellovibrionia Bacteriovoracales Bacteriovoracaceae Peredibacter ASV353 | Bacteria Proteobacteria Gammaproteobacteria Enterobacterales Colwelliaceae Thalassotalea ASV349 |
| Bacteria Proteobacteria Alphaproteobacteria Micavibrionales Micavibrionaceae ASV359 ASV359 | Bacteria Bdellovibrionota Bdellovibrionia Bacteriovoracales Bacteriovoracaceae Peredibacter ASV353 |
| Bacteria Bdellovibrionota Bdellovibrionia Bdellovibrionales Bdellovibrionaceae OM27 clade ASV371 | Bacteria Proteobacteria Alphaproteobacteria Micavibrionales Micavibrionaceae ASV359 ASV359 |
| Bacteria Proteobacteria Gammaproteobacteria Enterobacterales Kangiellaceae Kangiella ASV397 | Bacteria Bdellovibrionota Bdellovibrionia Bdellovibrionales Bdellovibrionaceae OM27 clade ASV371 |
| Bacteria Proteobacteria Alphaproteobacteria Rickettsiales AB1 ASV401 ASV401 | Bacteria Proteobacteria Gammaproteobacteria Enterobacterales Kangiellaceae Kangiella ASV397 |
| Bacteria Proteobacteria Alphaproteobacteria Caulobacterales Hyphomonadaceae ASV422 ASV422 | Bacteria Proteobacteria Alphaproteobacteria Rickettsiales AB1 ASV401 ASV401 |
| Bacteria Bacteroidota Bacteroidia Flavobacteriales Crocinitomicaceae Salinirepens ASV439 | Bacteria Proteobacteria Alphaproteobacteria Caulobacterales Hyphomonadaceae ASV422 ASV422 |
| Bacteria Bacteroidota Bacteroidia Flavobacteriales Flavobacteriaceae Kordia ASV441 | Bacteria Bacteroidota Bacteroidia Flavobacteriales Crocinitomicaceae Salinirepens ASV439 |
| Bacteria Proteobacteria Gammaproteobacteria Pseudomonadales Oleiphilaceae Oleiphilus ASV446 | Bacteria Bacteroidota Bacteroidia Flavobacteriales Flavobacteriaceae Kordia ASV441 |
| Bacteria Proteobacteria Gammaproteobacteria Enterobacterales Colwelliaceae Thalassotalea ASV455 | Bacteria Proteobacteria Gammaproteobacteria Pseudomonadales Oleiphilaceae Oleiphilus ASV446 |
| Bacteria Bacteroidota Bacteroidia Chitinophagales Saprospiraceae Lewinella ASV473 | Bacteria Proteobacteria Gammaproteobacteria Enterobacterales Colwelliaceae Thalassotalea ASV455 |
| Bacteria Proteobacteria Alphaproteobacteria Caulobacterales Hyphomonadaceae ASV481 ASV481 | Bacteria Bacteroidota Bacteroidia Chitinophagales Saprospiraceae Lewinella ASV473 |
| Bacteria Proteobacteria Alphaproteobacteria Rickettsiales Fokiniaceae ASV499 ASV499 | Bacteria Proteobacteria Alphaproteobacteria Caulobacterales Hyphomonadaceae ASV481 ASV481 |
| Bacteria Bacteroidota Bacteroidia Flavobacteriales Schleiferiaceae Schleiferia ASV511 | Bacteria Proteobacteria Alphaproteobacteria Rickettsiales Fokiniaceae ASV499 ASV499 |
| Bacteria Proteobacteria Alphaproteobacteria Caulobacterales Hyphomonadaceae Hyphomonas ASV522 | Bacteria Bacteroidota Bacteroidia Flavobacteriales Schleiferiaceae Schleiferia ASV511 |
| Bacteria Bacteroidota Bacteroidia Flavobacteriales Crocinitomicaceae Crocinitomix ASV539 | Bacteria Proteobacteria Alphaproteobacteria Caulobacterales Hyphomonadaceae Hyphomonas ASV522 |
| Bacteria Bacteroidota Bacteroidia Flavobacteriales Cryomorphaceae ASV541 ASV541 | Bacteria Bacteroidota Bacteroidia Flavobacteriales Crocinitomicaceae Crocinitomix ASV539 |
| Bacteria Proteobacteria Gammaproteobacteria Pseudomonadales Halieaceae OM60(NOR5) clade ASV546 | Bacteria Bacteroidota Bacteroidia Flavobacteriales Cryomorphaceae ASV541 ASV541 |
| Bacteria Proteobacteria Gammaproteobacteria Pseudomonadales Saccharospirillaceae ASV547 ASV547 | Bacteria Proteobacteria Gammaproteobacteria Pseudomonadales Halieaceae OM60(NOR5) clade ASV546 |
| Bacteria Bacteroidota Bacteroidia Flavobacteriales Flavobacteriaceae Mesoflavibacter ASV550 | Bacteria Proteobacteria Gammaproteobacteria Pseudomonadales Saccharospirillaceae ASV547 ASV547 |
| Bacteria Proteobacteria Gammaproteobacteria Pseudomonadales Saccharospirillaceae Oleibacter ASV556 | Bacteria Bacteroidota Bacteroidia Flavobacteriales Flavobacteriaceae Mesoflavibacter ASV550 |
| Bacteria Bacteroidota Bacteroidia Chitinophagales Saprospiraceae Lewinella ASV558 | Bacteria Proteobacteria Gammaproteobacteria Pseudomonadales Saccharospirillaceae Oleibacter ASV556 |
| Bacteria Bacteroidota Bacteroidia Flavobacteriales Crocinitomicaceae Crocinitomix ASV562 | Bacteria Bacteroidota Bacteroidia Chitinophagales Saprospiraceae Lewinella ASV558 |
| Bacteria Proteobacteria Alphaproteobacteria Rhodobacterales Rhodobacteraceae Pseudophaeobacter ASV584 | Bacteria Bacteroidota Bacteroidia Flavobacteriales Crocinitomicaceae Crocinitomix ASV562 |
| Bacteria Proteobacteria Alphaproteobacteria Micavibrionales Micavibrionaceae ASV606 ASV606 | Bacteria Proteobacteria Alphaproteobacteria Rhodobacterales Rhodobacteraceae Pseudophaeobacter ASV584 |
| Bacteria Proteobacteria Gammaproteobacteria Enterobacterales Alteromonadaceae ASV609 ASV609 | Bacteria Proteobacteria Alphaproteobacteria Micavibrionales Micavibrionaceae ASV606 ASV606 |
| Bacteria Proteobacteria Alphaproteobacteria Rhodobacterales Rhodobacteraceae Tropicibacter ASV655 | Bacteria Proteobacteria Gammaproteobacteria Enterobacterales Alteromonadaceae ASV609 ASV609 |
| Bacteria Bdellovibrionota Bdellovibrionia Bdellovibrionales Bdellovibrionaceae OM27 clade ASV686 | Bacteria Proteobacteria Alphaproteobacteria Rhodobacterales Rhodobacteraceae Tropicibacter ASV655 |
| Bacteria Bacteroidota Bacteroidia Flavobacteriales Cryomorphaceae ASV688 ASV688 | Bacteria Bdellovibrionota Bdellovibrionia Bdellovibrionales Bdellovibrionaceae OM27 clade ASV686 |
| Bacteria Proteobacteria Alphaproteobacteria Rickettsiales Rickettsiaceae Candidatus Megaira ASV700 | Bacteria Bacteroidota Bacteroidia Flavobacteriales Cryomorphaceae ASV688 ASV688 |
| Bacteria Proteobacteria Gammaproteobacteria Enterobacterales Colwelliaceae Thalassotalea ASV712 | Bacteria Proteobacteria Alphaproteobacteria Rickettsiales Rickettsiaceae Candidatus Megaira ASV700 |
| Bacteria Myxococcota Polyangia Nannocystales Nannocystaceae ASV713 ASV713 | Bacteria Proteobacteria Gammaproteobacteria Enterobacterales Colwelliaceae Thalassotalea ASV712 |
| Bacteria Bacteroidota Bacteroidia Cytophagales Cyclobacteriaceae Reichenbachiella ASV720 | Bacteria Myxococcota Polyangia Nannocystales Nannocystaceae ASV713 ASV713 |
| Bacteria Bacteroidota Bacteroidia Flavobacteriales Crocinitomicaceae Fluviicola ASV735 | Bacteria Bacteroidota Bacteroidia Cytophagales Cyclobacteriaceae Reichenbachiella ASV720 |
| Bacteria Proteobacteria Gammaproteobacteria Enterobacterales Alteromonadaceae Alteromonas ASV736 | Bacteria Bacteroidota Bacteroidia Flavobacteriales Crocinitomicaceae Fluviicola ASV735 |
| Bacteria Bacteroidota Bacteroidia Flavobacteriales Flavobacteriaceae Ulvibacter ASV738 | Bacteria Proteobacteria Gammaproteobacteria Enterobacterales Alteromonadaceae Alteromonas ASV736 |
| Bacteria Bacteroidota Bacteroidia Flavobacteriales Crocinitomicaceae Crocinitomix ASV744 | Bacteria Bacteroidota Bacteroidia Flavobacteriales Flavobacteriaceae Ulvibacter ASV738 |
| Bacteria Bdellovibrionota Bdellovibrionia Bdellovibrionales Bdellovibrionaceae OM27 clade ASV767 | Bacteria Bacteroidota Bacteroidia Flavobacteriales Crocinitomicaceae Crocinitomix ASV744 |
| Bacteria Bacteroidota Bacteroidia Cytophagales Cyclobacteriaceae ASV775 ASV775 | Bacteria Bdellovibrionota Bdellovibrionia Bdellovibrionales Bdellovibrionaceae OM27 clade ASV767 |
| Archaea Nanoarchaeota Nanoarchaeia Woesearchaeales SCGC AAA286-E23 ASV777 ASV777 | Bacteria Bacteroidota Bacteroidia Cytophagales Cyclobacteriaceae ASV775 ASV775 |
| Bacteria Proteobacteria Gammaproteobacteria Enterobacterales Kangiellaceae Aliikangiella ASV784 | Archaea Nanoarchaeota Nanoarchaeia Woesearchaeales SCGC AAA286-E23 ASV777 ASV777 |
| Bacteria Bacteroidota Bacteroidia Cytophagales Cyclobacteriaceae Marinoscillum ASV812 | Bacteria Proteobacteria Gammaproteobacteria Enterobacterales Kangiellaceae Aliikangiella ASV784 |
| Bacteria Bdellovibrionota Bdellovibrionia Bdellovibrionales Bdellovibrionaceae OM27 clade ASV833 | Bacteria Bacteroidota Bacteroidia Cytophagales Cyclobacteriaceae Marinoscillum ASV812 |
| Bacteria Proteobacteria Alphaproteobacteria Caulobacterales Hyphomonadaceae Oceanicaulis ASV854 | Bacteria Bdellovibrionota Bdellovibrionia Bdellovibrionales Bdellovibrionaceae OM27 clade ASV833 |
| Bacteria Proteobacteria Gammaproteobacteria Arenicellales Arenicellaceae Arenicella ASV875 | Bacteria Proteobacteria Alphaproteobacteria Caulobacterales Hyphomonadaceae Oceanicaulis ASV854 |
| Bacteria Bacteroidota Bacteroidia Flavobacteriales Flavobacteriaceae ASV882 ASV882 | Bacteria Proteobacteria Gammaproteobacteria Arenicellales Arenicellaceae Arenicella ASV875 |
| Bacteria Bacteroidota Rhodothermia Rhodothermales Rhodothermaceae ASV888 ASV888 | Bacteria Bacteroidota Bacteroidia Flavobacteriales Flavobacteriaceae ASV882 ASV882 |
| Bacteria Bdellovibrionota Bdellovibrionia Bdellovibrionales Bdellovibrionaceae OM27 clade ASV905 | Bacteria Bacteroidota Rhodothermia Rhodothermales Rhodothermaceae ASV888 ASV888 |
| Bacteria Proteobacteria Alphaproteobacteria Caulobacterales Hyphomonadaceae Ponticaulis ASV910 | Bacteria Bdellovibrionota Bdellovibrionia Bdellovibrionales Bdellovibrionaceae OM27 clade ASV905 |
| Bacteria Proteobacteria Gammaproteobacteria Pseudomonadales Oleiphilaceae Oleiphilus ASV911 | Bacteria Proteobacteria Alphaproteobacteria Caulobacterales Hyphomonadaceae Ponticaulis ASV910 |
| Bacteria Myxococcota Myxococcia Myxococcales Myxococcaceae P3OB-42 ASV919 | Bacteria Proteobacteria Gammaproteobacteria Pseudomonadales Oleiphilaceae Oleiphilus ASV911 |
| Bacteria Proteobacteria Alphaproteobacteria Micavibrionales Micavibrionaceae ASV922 ASV922 | Bacteria Myxococcota Myxococcia Myxococcales Myxococcaceae P3OB-42 ASV919 |
| Bacteria Proteobacteria Alphaproteobacteria Caulobacterales Hyphomonadaceae Maricaulis ASV958 | Bacteria Proteobacteria Alphaproteobacteria Micavibrionales Micavibrionaceae ASV922 ASV922 |
| Bacteria Proteobacteria Gammaproteobacteria Pseudomonadales Spongiibacteraceae BD1-7 clade ASV979 | Bacteria Proteobacteria Alphaproteobacteria Caulobacterales Hyphomonadaceae Maricaulis ASV958 |
| Bacteria Bacteroidota Bacteroidia Chitinophagales Saprospiraceae ASV987 ASV987 | Bacteria Proteobacteria Gammaproteobacteria Pseudomonadales Spongiibacteraceae BD1-7 clade ASV979 |
| Bacteria Bacteroidota Bacteroidia Chitinophagales Saprospiraceae Aureispira ASV994 | Bacteria Bacteroidota Bacteroidia Chitinophagales Saprospiraceae ASV987 ASV987 |
| Bacteria Bdellovibrionota Bdellovibrionia Bdellovibrionales Bdellovibrionaceae OM27 clade ASV1004 | Bacteria Bacteroidota Bacteroidia Chitinophagales Saprospiraceae Aureispira ASV994 |
| Bacteria Bacteroidota Bacteroidia Cytophagales Cyclobacteriaceae Reichenbachiella ASV1014 | Bacteria Bdellovibrionota Bdellovibrionia Bdellovibrionales Bdellovibrionaceae OM27 clade ASV1004 |
| Bacteria Bacteroidota Bacteroidia Cytophagales Cyclobacteriaceae Fabibacter ASV1054 | Bacteria Bacteroidota Bacteroidia Cytophagales Cyclobacteriaceae Reichenbachiella ASV1014 |
| Bacteria Bacteroidota Bacteroidia Flavobacteriales Flavobacteriaceae ASV1062 ASV1062 | Bacteria Bacteroidota Bacteroidia Cytophagales Cyclobacteriaceae Fabibacter ASV1054 |
| Bacteria Bacteroidota Bacteroidia Flavobacteriales Flavobacteriaceae Tenacibaculum ASV1075 | Bacteria Bacteroidota Bacteroidia Flavobacteriales Flavobacteriaceae ASV1062 ASV1062 |
| Bacteria Bdellovibrionota Bdellovibrionia Bacteriovoracales Bacteriovoracaceae Halobacteriovorax ASV1099 | Bacteria Bacteroidota Bacteroidia Flavobacteriales Flavobacteriaceae Tenacibaculum ASV1075 |
| Bacteria Proteobacteria Alphaproteobacteria Caulobacterales Hyphomonadaceae ASV1123 ASV1123 | Bacteria Bdellovibrionota Bdellovibrionia Bacteriovoracales Bacteriovoracaceae Halobacteriovorax ASV1099 |
| Bacteria Bacteroidota Bacteroidia Flavobacteriales Cryomorphaceae ASV1168 ASV1168 | Bacteria Proteobacteria Alphaproteobacteria Caulobacterales Hyphomonadaceae ASV1123 ASV1123 |
| Bacteria Proteobacteria Alphaproteobacteria Rhizobiales Rhizobiaceae Cohaesibacter ASV1198 | Bacteria Bacteroidota Bacteroidia Flavobacteriales Cryomorphaceae ASV1168 ASV1168 |
| Bacteria Proteobacteria Gammaproteobacteria Enterobacterales Alteromonadaceae Alteromonas ASV1214 | Bacteria Proteobacteria Alphaproteobacteria Rhizobiales Rhizobiaceae Cohaesibacter ASV1198 |
| Bacteria Proteobacteria Gammaproteobacteria Enterobacterales Alteromonadaceae Alteromonas ASV1227 | Bacteria Proteobacteria Gammaproteobacteria Enterobacterales Alteromonadaceae Alteromonas ASV1214 |
| Bacteria Proteobacteria Alphaproteobacteria Sphingomonadales Sphingomonadaceae ASV1235 ASV1235 | Bacteria Proteobacteria Gammaproteobacteria Enterobacterales Alteromonadaceae Alteromonas ASV1227 |
| Bacteria Bacteroidota Bacteroidia Flavobacteriales Cryomorphaceae ASV1241 ASV1241 | Bacteria Proteobacteria Alphaproteobacteria Sphingomonadales Sphingomonadaceae ASV1235 ASV1235 |
| Bacteria Bacteroidota Bacteroidia Flavobacteriales Cryomorphaceae ASV1250 ASV1250 | Bacteria Bacteroidota Bacteroidia Flavobacteriales Cryomorphaceae ASV1241 ASV1241 |
| Bacteria Proteobacteria Gammaproteobacteria Pseudomonadales Nitrincolaceae Profundimonas ASV1259 | Bacteria Bacteroidota Bacteroidia Flavobacteriales Cryomorphaceae ASV1250 ASV1250 |
| Bacteria Bacteroidota Bacteroidia Flavobacteriales Cryomorphaceae Vicingus ASV1266 | Bacteria Proteobacteria Gammaproteobacteria Pseudomonadales Nitrincolaceae Profundimonas ASV1259 |
| Bacteria Proteobacteria Alphaproteobacteria Rhodobacterales Rhodobacteraceae Cognatishimia ASV1289 | Bacteria Bacteroidota Bacteroidia Flavobacteriales Cryomorphaceae Vicingus ASV1266 |
| Bacteria Bacteroidota Bacteroidia Chitinophagales Saprospiraceae Rubidimonas ASV1295 | Bacteria Proteobacteria Alphaproteobacteria Rhodobacterales Rhodobacteraceae Cognatishimia ASV1289 |
| Bacteria Bacteroidota Bacteroidia Chitinophagales Saprospiraceae Saprospira ASV1297 | Bacteria Bacteroidota Bacteroidia Chitinophagales Saprospiraceae Rubidimonas ASV1295 |
| Bacteria Proteobacteria Gammaproteobacteria Enterobacterales Colwelliaceae Thalassotalea ASV1299 | Bacteria Bacteroidota Bacteroidia Chitinophagales Saprospiraceae Saprospira ASV1297 |
| Bacteria Dependentiae Babeliae Babeliales UBA12411 ASV1305 ASV1305 | Bacteria Proteobacteria Gammaproteobacteria Enterobacterales Colwelliaceae Thalassotalea ASV1299 |
| Bacteria Proteobacteria Gammaproteobacteria Enterobacterales Colwelliaceae Thalassotalea ASV1311 | Bacteria Dependentiae Babeliae Babeliales UBA12411 ASV1305 ASV1305 |
| Bacteria Bacteroidota Bacteroidia Flavobacteriales Cryomorphaceae Vicingus ASV1321 | Bacteria Proteobacteria Gammaproteobacteria Enterobacterales Colwelliaceae Thalassotalea ASV1311 |
| Bacteria Bacteroidota Bacteroidia Cytophagales Cyclobacteriaceae Ekhidna ASV1324 | Bacteria Bacteroidota Bacteroidia Flavobacteriales Cryomorphaceae Vicingus ASV1321 |
| Bacteria Proteobacteria Gammaproteobacteria Enterobacterales Shewanellaceae Psychrobium ASV1377 | Bacteria Bacteroidota Bacteroidia Cytophagales Cyclobacteriaceae Ekhidna ASV1324 |
| Bacteria Bacteroidota Bacteroidia Flavobacteriales Cryomorphaceae Owenweeksia ASV1391 | Bacteria Proteobacteria Gammaproteobacteria Enterobacterales Shewanellaceae Psychrobium ASV1377 |
| Bacteria Proteobacteria Gammaproteobacteria Pseudomonadales Oleiphilaceae Oleiphilus ASV1408 | Bacteria Bacteroidota Bacteroidia Flavobacteriales Cryomorphaceae Owenweeksia ASV1391 |
| Bacteria Proteobacteria Gammaproteobacteria Pseudomonadales Oleiphilaceae Oleiphilus ASV1421 | Bacteria Proteobacteria Gammaproteobacteria Pseudomonadales Oleiphilaceae Oleiphilus ASV1408 |
| Bacteria Proteobacteria Gammaproteobacteria Pseudomonadales Spongiibacteraceae BD1-7 clade ASV1445 | Bacteria Proteobacteria Gammaproteobacteria Pseudomonadales Oleiphilaceae Oleiphilus ASV1421 |
| Bacteria Proteobacteria Alphaproteobacteria Micavibrionales Micavibrionaceae ASV1453 ASV1453 | Bacteria Proteobacteria Gammaproteobacteria Pseudomonadales Spongiibacteraceae BD1-7 clade ASV1445 |
| Bacteria Proteobacteria Alphaproteobacteria Rhizobiales Rhizobiaceae Pseudahrensia ASV1459 | Bacteria Proteobacteria Alphaproteobacteria Micavibrionales Micavibrionaceae ASV1453 ASV1453 |
| Bacteria Bdellovibrionota Bdellovibrionia Bdellovibrionales Bdellovibrionaceae OM27 clade ASV1470 | Bacteria Proteobacteria Alphaproteobacteria Rhizobiales Rhizobiaceae Pseudahrensia ASV1459 |
| Bacteria Bacteroidota Bacteroidia Flavobacteriales Flavobacteriaceae Winogradskyella ASV1474 | Bacteria Bdellovibrionota Bdellovibrionia Bdellovibrionales Bdellovibrionaceae OM27 clade ASV1470 |
| Bacteria Bacteroidota Bacteroidia Bacteroidales Marinilabiliaceae Carboxylicivirga ASV1477 | Bacteria Bacteroidota Bacteroidia Flavobacteriales Flavobacteriaceae Winogradskyella ASV1474 |
| Bacteria Bacteroidota Bacteroidia Flavobacteriales NS9 marine group ASV1478 ASV1478 | Bacteria Bacteroidota Bacteroidia Bacteroidales Marinilabiliaceae Carboxylicivirga ASV1477 |
| Bacteria Proteobacteria Gammaproteobacteria Pseudomonadales P13-46 ASV1498 ASV1498 | Bacteria Bacteroidota Bacteroidia Flavobacteriales NS9 marine group ASV1478 ASV1478 |
| Bacteria Bdellovibrionota Bdellovibrionia Bdellovibrionales Bdellovibrionaceae OM27 clade ASV1503 | Bacteria Proteobacteria Gammaproteobacteria Pseudomonadales P13-46 ASV1498 ASV1498 |
| Bacteria Proteobacteria Alphaproteobacteria Caulobacterales Hyphomonadaceae Algimonas ASV1533 | Bacteria Bdellovibrionota Bdellovibrionia Bdellovibrionales Bdellovibrionaceae OM27 clade ASV1503 |
| Bacteria Proteobacteria Gammaproteobacteria Enterobacterales Colwelliaceae Thalassotalea ASV1543 | Bacteria Proteobacteria Alphaproteobacteria Caulobacterales Hyphomonadaceae Algimonas ASV1533 |
| Bacteria Bdellovibrionota Bdellovibrionia Bacteriovoracales Bacteriovoracaceae Peredibacter ASV1627 | Bacteria Proteobacteria Gammaproteobacteria Enterobacterales Colwelliaceae Thalassotalea ASV1543 |
| Bacteria Proteobacteria Gammaproteobacteria Enterobacterales Shewanellaceae Ferrimonas ASV1637 | Bacteria Bdellovibrionota Bdellovibrionia Bacteriovoracales Bacteriovoracaceae Peredibacter ASV1627 |
| Bacteria Bacteroidota Bacteroidia Flavobacteriales Cryomorphaceae Vicingus ASV1657 | Bacteria Proteobacteria Gammaproteobacteria Enterobacterales Shewanellaceae Ferrimonas ASV1637 |
| Bacteria Bdellovibrionota Bdellovibrionia Bdellovibrionales Bdellovibrionaceae OM27 clade ASV1702 | Bacteria Bacteroidota Bacteroidia Flavobacteriales Cryomorphaceae Vicingus ASV1657 |
| Bacteria Proteobacteria Alphaproteobacteria Caulobacterales Hyphomonadaceae ASV1762 ASV1762 | Bacteria Bdellovibrionota Bdellovibrionia Bdellovibrionales Bdellovibrionaceae OM27 clade ASV1702 |
| Bacteria Bacteroidota Bacteroidia Flavobacteriales Crocinitomicaceae Lishizhenia ASV1768 | Bacteria Proteobacteria Alphaproteobacteria Caulobacterales Hyphomonadaceae ASV1762 ASV1762 |
| Bacteria Bacteroidota Bacteroidia Flavobacteriales Cryomorphaceae Vicingus ASV1772 | Bacteria Bacteroidota Bacteroidia Flavobacteriales Crocinitomicaceae Lishizhenia ASV1768 |
| Bacteria Planctomycetota Phycisphaerae Phycisphaerales Phycisphaeraceae SM1A02 ASV1817 | Bacteria Bacteroidota Bacteroidia Flavobacteriales Cryomorphaceae Vicingus ASV1772 |
| Bacteria Bacteroidota Bacteroidia Flavobacteriales Flavobacteriaceae ASV1846 ASV1846 | Bacteria Planctomycetota Phycisphaerae Phycisphaerales Phycisphaeraceae SM1A02 ASV1817 |
| Bacteria Bdellovibrionota Bdellovibrionia Bacteriovoracales Bacteriovoracaceae Peredibacter ASV1868 | Bacteria Bacteroidota Bacteroidia Flavobacteriales Flavobacteriaceae ASV1846 ASV1846 |
| Bacteria Proteobacteria Alphaproteobacteria Caulobacterales Hyphomonadaceae Maricaulis ASV1924 | Bacteria Bdellovibrionota Bdellovibrionia Bacteriovoracales Bacteriovoracaceae Peredibacter ASV1868 |
| Bacteria Bacteroidota Bacteroidia Flavobacteriales Cryomorphaceae ASV1936 ASV1936 | Bacteria Proteobacteria Alphaproteobacteria Caulobacterales Hyphomonadaceae Maricaulis ASV1924 |
| Bacteria Bacteroidota Bacteroidia Flavobacteriales Flavobacteriaceae Kordia ASV1938 | Bacteria Bacteroidota Bacteroidia Flavobacteriales Cryomorphaceae ASV1936 ASV1936 |
| Bacteria Proteobacteria Alphaproteobacteria Caulobacterales Hyphomonadaceae Hellea ASV1941 | Bacteria Bacteroidota Bacteroidia Flavobacteriales Flavobacteriaceae Kordia ASV1938 |
| Bacteria Bacteroidota Bacteroidia Chitinophagales Saprospiraceae ASV1965 ASV1965 | Bacteria Proteobacteria Alphaproteobacteria Caulobacterales Hyphomonadaceae Hellea ASV1941 |
| Bacteria Proteobacteria Gammaproteobacteria Pseudomonadales Nitrincolaceae Neptuniibacter ASV1971 | Bacteria Bacteroidota Bacteroidia Chitinophagales Saprospiraceae ASV1965 ASV1965 |
| Bacteria Proteobacteria Gammaproteobacteria Pseudomonadales Saccharospirillaceae Reinekea ASV1977 | Bacteria Proteobacteria Gammaproteobacteria Pseudomonadales Nitrincolaceae Neptuniibacter ASV1971 |
| Bacteria Bdellovibrionota Bdellovibrionia Bdellovibrionales Bdellovibrionaceae OM27 clade ASV1986 | Bacteria Proteobacteria Gammaproteobacteria Pseudomonadales Saccharospirillaceae Reinekea ASV1977 |
| Bacteria Bacteroidota Bacteroidia Flavobacteriales Crocinitomicaceae Salinirepens ASV2005 | Bacteria Bdellovibrionota Bdellovibrionia Bdellovibrionales Bdellovibrionaceae OM27 clade ASV1986 |
| Bacteria Proteobacteria Gammaproteobacteria Pseudomonadales Nitrincolaceae Marinobacterium ASV2021 | Bacteria Bacteroidota Bacteroidia Flavobacteriales Crocinitomicaceae Salinirepens ASV2005 |
| Bacteria Proteobacteria Alphaproteobacteria Rhodobacterales Rhodobacteraceae ASV2066 ASV2066 | Bacteria Proteobacteria Gammaproteobacteria Pseudomonadales Nitrincolaceae Marinobacterium ASV2021 |
| Bacteria Proteobacteria Alphaproteobacteria Caulobacterales Hyphomonadaceae Oceanicaulis ASV2130 | Bacteria Proteobacteria Alphaproteobacteria Rhodobacterales Rhodobacteraceae ASV2066 ASV2066 |
| Bacteria Bacteroidota Bacteroidia Flavobacteriales Crocinitomicaceae ASV2142 ASV2142 | Bacteria Proteobacteria Alphaproteobacteria Caulobacterales Hyphomonadaceae Oceanicaulis ASV2130 |
| Bacteria Bdellovibrionota Bdellovibrionia Bdellovibrionales Bdellovibrionaceae OM27 clade ASV2168 | Bacteria Bacteroidota Bacteroidia Flavobacteriales Crocinitomicaceae ASV2142 ASV2142 |
| Bacteria Proteobacteria Gammaproteobacteria Arenicellales Arenicellaceae HTCC5015 ASV2178 | Bacteria Bdellovibrionota Bdellovibrionia Bdellovibrionales Bdellovibrionaceae OM27 clade ASV2168 |
| Bacteria Bdellovibrionota Bdellovibrionia Bacteriovoracales Bacteriovoracaceae Halobacteriovorax ASV2228 | Bacteria Proteobacteria Gammaproteobacteria Arenicellales Arenicellaceae HTCC5015 ASV2178 |
| Bacteria Myxococcota Polyangia Polyangiales Sandaracinaceae ASV2235 ASV2235 | Bacteria Bdellovibrionota Bdellovibrionia Bacteriovoracales Bacteriovoracaceae Halobacteriovorax ASV2228 |
| Bacteria Bacteroidota Bacteroidia Flavobacteriales Flavobacteriaceae Tenacibaculum ASV2240 | Bacteria Myxococcota Polyangia Polyangiales Sandaracinaceae ASV2235 ASV2235 |
| Bacteria Bacteroidota Bacteroidia Chitinophagales Saprospiraceae ASV2263 ASV2263 | Bacteria Bacteroidota Bacteroidia Flavobacteriales Flavobacteriaceae Tenacibaculum ASV2240 |
| Bacteria Bacteroidota Bacteroidia Flavobacteriales Crocinitomicaceae Fluviicola ASV2266 | Bacteria Bacteroidota Bacteroidia Chitinophagales Saprospiraceae ASV2263 ASV2263 |
| Bacteria Proteobacteria Gammaproteobacteria Arenicellales Arenicellaceae HTCC5015 ASV2305 | Bacteria Bacteroidota Bacteroidia Flavobacteriales Crocinitomicaceae Fluviicola ASV2266 |
| Bacteria Proteobacteria Alphaproteobacteria Caulobacterales Hyphomonadaceae Oceanicaulis ASV2307 | Bacteria Proteobacteria Gammaproteobacteria Arenicellales Arenicellaceae HTCC5015 ASV2305 |
| Bacteria Proteobacteria Gammaproteobacteria Enterobacterales Alteromonadaceae Aliiglaciecola ASV2357 | Bacteria Proteobacteria Alphaproteobacteria Caulobacterales Hyphomonadaceae Oceanicaulis ASV2307 |
| Bacteria Bacteroidota Bacteroidia Flavobacteriales Crocinitomicaceae Crocinitomix ASV2363 | Bacteria Proteobacteria Gammaproteobacteria Enterobacterales Alteromonadaceae Aliiglaciecola ASV2357 |
| Bacteria Bacteroidota Bacteroidia Flavobacteriales Cryomorphaceae ASV2372 ASV2372 | Bacteria Bacteroidota Bacteroidia Flavobacteriales Crocinitomicaceae Crocinitomix ASV2363 |
| Bacteria Proteobacteria Alphaproteobacteria Micavibrionales Micavibrionaceae ASV2402 ASV2402 | Bacteria Bacteroidota Bacteroidia Flavobacteriales Cryomorphaceae ASV2372 ASV2372 |
| Bacteria Bacteroidota Bacteroidia Flavobacteriales Flavobacteriaceae Pseudofulvibacter ASV2403 | Bacteria Proteobacteria Alphaproteobacteria Micavibrionales Micavibrionaceae ASV2402 ASV2402 |
| Bacteria Bdellovibrionota Bdellovibrionia Bdellovibrionales Bdellovibrionaceae OM27 clade ASV2487 | Bacteria Bacteroidota Bacteroidia Flavobacteriales Flavobacteriaceae Pseudofulvibacter ASV2403 |
| Bacteria Proteobacteria Alphaproteobacteria Micavibrionales Micavibrionaceae ASV2528 ASV2528 | Bacteria Bdellovibrionota Bdellovibrionia Bdellovibrionales Bdellovibrionaceae OM27 clade ASV2487 |
| Archaea Nanoarchaeota Nanoarchaeia Woesearchaeales SCGC AAA286-E23 ASV2574 ASV2574 | Bacteria Proteobacteria Alphaproteobacteria Micavibrionales Micavibrionaceae ASV2528 ASV2528 |
| Bacteria Bdellovibrionota Bdellovibrionia Bdellovibrionales Bdellovibrionaceae OM27 clade ASV2661 | Archaea Nanoarchaeota Nanoarchaeia Woesearchaeales SCGC AAA286-E23 ASV2574 ASV2574 |
| Bacteria Proteobacteria Gammaproteobacteria Salinisphaerales Solimonadaceae Oceanococcus ASV2662 | Bacteria Bdellovibrionota Bdellovibrionia Bdellovibrionales Bdellovibrionaceae OM27 clade ASV2661 |
| Bacteria Bacteroidota Bacteroidia Flavobacteriales Cryomorphaceae Owenweeksia ASV2669 | Bacteria Proteobacteria Gammaproteobacteria Salinisphaerales Solimonadaceae Oceanococcus ASV2662 |
| Bacteria Proteobacteria Alphaproteobacteria Caulobacterales Hyphomonadaceae Algimonas ASV2691 | Bacteria Bacteroidota Bacteroidia Flavobacteriales Cryomorphaceae Owenweeksia ASV2669 |
| Bacteria Bdellovibrionota Bdellovibrionia Bdellovibrionales Bdellovibrionaceae OM27 clade ASV2692 | Bacteria Proteobacteria Alphaproteobacteria Caulobacterales Hyphomonadaceae Algimonas ASV2691 |
| Bacteria Bacteroidota Bacteroidia Flavobacteriales Flavobacteriaceae Nonlabens ASV2698 | Bacteria Bdellovibrionota Bdellovibrionia Bdellovibrionales Bdellovibrionaceae OM27 clade ASV2692 |
| Bacteria Proteobacteria Gammaproteobacteria Pseudomonadales Spongiibacteraceae BD1-7 clade ASV2730 | Bacteria Bacteroidota Bacteroidia Flavobacteriales Flavobacteriaceae Nonlabens ASV2698 |
| Bacteria Bacteroidota Bacteroidia Cytophagales Flammeovirgaceae Flammeovirga ASV2762 | Bacteria Proteobacteria Gammaproteobacteria Pseudomonadales Spongiibacteraceae BD1-7 clade ASV2730 |
| Bacteria Proteobacteria Gammaproteobacteria Arenicellales Arenicellaceae HTCC5015 ASV2823 | Bacteria Bacteroidota Bacteroidia Cytophagales Flammeovirgaceae Flammeovirga ASV2762 |
| Bacteria Firmicutes Bacilli Mycoplasmatales Mycoplasmataceae Candidatus Bacilloplasma ASV2834 | Bacteria Proteobacteria Gammaproteobacteria Arenicellales Arenicellaceae HTCC5015 ASV2823 |
| Bacteria Proteobacteria Alphaproteobacteria Caulobacterales Hyphomonadaceae Maricaulis ASV2841 | Bacteria Firmicutes Bacilli Mycoplasmatales Mycoplasmataceae Candidatus Bacilloplasma ASV2834 |
| Bacteria Bdellovibrionota Bdellovibrionia Bacteriovoracales Bacteriovoracaceae Peredibacter ASV2942 | Bacteria Proteobacteria Alphaproteobacteria Caulobacterales Hyphomonadaceae Maricaulis ASV2841 |
| Bacteria Planctomycetota Phycisphaerae Phycisphaerales Phycisphaeraceae SM1A02 ASV2976 | Bacteria Bdellovibrionota Bdellovibrionia Bacteriovoracales Bacteriovoracaceae Peredibacter ASV2942 |
| Bacteria Proteobacteria Gammaproteobacteria Pseudomonadales Cellvibrionaceae Pseudomaricurvus ASV2998 | Bacteria Planctomycetota Phycisphaerae Phycisphaerales Phycisphaeraceae SM1A02 ASV2976 |
| Bacteria Bdellovibrionota Bdellovibrionia Bdellovibrionales Bdellovibrionaceae OM27 clade ASV3057 | Bacteria Proteobacteria Gammaproteobacteria Pseudomonadales Cellvibrionaceae Pseudomaricurvus ASV2998 |
| Bacteria Proteobacteria Gammaproteobacteria Enterobacterales Alteromonadaceae Neiella ASV3059 | Bacteria Bdellovibrionota Bdellovibrionia Bdellovibrionales Bdellovibrionaceae OM27 clade ASV3057 |
| Bacteria Proteobacteria Gammaproteobacteria Pseudomonadales Nitrincolaceae Amphritea ASV3116 | Bacteria Proteobacteria Gammaproteobacteria Enterobacterales Alteromonadaceae Neiella ASV3059 |
| Bacteria Proteobacteria Alphaproteobacteria Micavibrionales Micavibrionaceae ASV3184 ASV3184 | Bacteria Proteobacteria Gammaproteobacteria Pseudomonadales Nitrincolaceae Amphritea ASV3116 |
| Bacteria Proteobacteria Alphaproteobacteria Sphingomonadales Sphingomonadaceae Erythrobacter ASV3234 | Bacteria Proteobacteria Alphaproteobacteria Micavibrionales Micavibrionaceae ASV3184 ASV3184 |
| Bacteria Proteobacteria Gammaproteobacteria Enterobacterales Pseudoalteromonadaceae Psychrosphaera ASV3291 | Bacteria Proteobacteria Alphaproteobacteria Sphingomonadales Sphingomonadaceae Erythrobacter ASV3234 |
| Bacteria Bacteroidota Bacteroidia Flavobacteriales Flavobacteriaceae Tenacibaculum ASV3436 | Bacteria Proteobacteria Gammaproteobacteria Enterobacterales Pseudoalteromonadaceae Psychrosphaera ASV3291 |
| Bacteria Proteobacteria Gammaproteobacteria Burkholderiales Burkholderiaceae Limnobacter ASV3471 | Bacteria Bacteroidota Bacteroidia Flavobacteriales Flavobacteriaceae Tenacibaculum ASV3436 |
| Bacteria Proteobacteria Alphaproteobacteria Caulobacterales Parvularculaceae ASV3484 ASV3484 | Bacteria Proteobacteria Gammaproteobacteria Burkholderiales Burkholderiaceae Limnobacter ASV3471 |
| Bacteria Bacteroidota Bacteroidia Sphingobacteriales NS11-12 marine group ASV3504 ASV3504 | Bacteria Proteobacteria Alphaproteobacteria Caulobacterales Parvularculaceae ASV3484 ASV3484 |
| Bacteria Bacteroidota Bacteroidia Flavobacteriales Crocinitomicaceae Salinirepens ASV3644 | Bacteria Bacteroidota Bacteroidia Sphingobacteriales NS11-12 marine group ASV3504 ASV3504 |
| Bacteria Proteobacteria Gammaproteobacteria Pseudomonadales Hahellaceae Hahella ASV3844 | Bacteria Bacteroidota Bacteroidia Flavobacteriales Crocinitomicaceae Salinirepens ASV3644 |
| Bacteria Bacteroidota Bacteroidia Flavobacteriales Crocinitomicaceae Fluviicola ASV3951 | Bacteria Proteobacteria Gammaproteobacteria Pseudomonadales Hahellaceae Hahella ASV3844 |
| Bacteria Bdellovibrionota Bdellovibrionia Bdellovibrionales Bdellovibrionaceae OM27 clade ASV3998 | Bacteria Bacteroidota Bacteroidia Flavobacteriales Crocinitomicaceae Fluviicola ASV3951 |
| Bacteria Proteobacteria Gammaproteobacteria Enterobacterales Colwelliaceae Thalassotalea ASV4071 | Bacteria Bdellovibrionota Bdellovibrionia Bdellovibrionales Bdellovibrionaceae OM27 clade ASV3998 |
| Bacteria Bacteroidota Bacteroidia Flavobacteriales Crocinitomicaceae Crocinitomix ASV4108 | Bacteria Proteobacteria Gammaproteobacteria Enterobacterales Colwelliaceae Thalassotalea ASV4071 |
| Bacteria Proteobacteria Alphaproteobacteria Micavibrionales Micavibrionaceae ASV4122 ASV4122 | Bacteria Bacteroidota Bacteroidia Flavobacteriales Crocinitomicaceae Crocinitomix ASV4108 |
| Bacteria Bacteroidota Bacteroidia Cytophagales Cyclobacteriaceae Algoriphagus ASV4123 | Bacteria Proteobacteria Alphaproteobacteria Micavibrionales Micavibrionaceae ASV4122 ASV4122 |
| Bacteria Proteobacteria Alphaproteobacteria Rickettsiales AB1 ASV4181 ASV4181 | Bacteria Bacteroidota Bacteroidia Cytophagales Cyclobacteriaceae Algoriphagus ASV4123 |
| Bacteria Proteobacteria Gammaproteobacteria Thiotrichales Thiotrichaceae Thiothrix ASV4466 | Bacteria Proteobacteria Alphaproteobacteria Rickettsiales AB1 ASV4181 ASV4181 |
| Bacteria Proteobacteria Alphaproteobacteria Micavibrionales Micavibrionaceae ASV4496 ASV4496 | Bacteria Proteobacteria Gammaproteobacteria Thiotrichales Thiotrichaceae Thiothrix ASV4466 |
| Bacteria Proteobacteria Alphaproteobacteria Paracaedibacterales Paracaedibacteraceae Candidatus Captivus ASV4497 | Bacteria Proteobacteria Alphaproteobacteria Micavibrionales Micavibrionaceae ASV4496 ASV4496 |
| Bacteria Bacteroidota Bacteroidia Flavobacteriales Flavobacteriaceae ASV4590 ASV4590 | Bacteria Proteobacteria Alphaproteobacteria Paracaedibacterales Paracaedibacteraceae Candidatus Captivus ASV4497 |
| Bacteria Proteobacteria Gammaproteobacteria Pseudomonadales Nitrincolaceae Marinobacterium ASV4591 | Bacteria Bacteroidota Bacteroidia Flavobacteriales Flavobacteriaceae ASV4590 ASV4590 |
| Bacteria Proteobacteria Gammaproteobacteria Granulosicoccales Granulosicoccaceae Granulosicoccus ASV5159 | Bacteria Proteobacteria Gammaproteobacteria Pseudomonadales Nitrincolaceae Marinobacterium ASV4591 |
| Bacteria Bacteroidota Bacteroidia Flavobacteriales Flavobacteriaceae Muricauda ASV5510 | Bacteria Proteobacteria Gammaproteobacteria Granulosicoccales Granulosicoccaceae Granulosicoccus ASV5159 |
| Bacteria Bacteroidota Bacteroidia Chitinophagales Saprospiraceae Aureispira ASV5511 | Bacteria Bacteroidota Bacteroidia Flavobacteriales Flavobacteriaceae Muricauda ASV5510 |
| Bacteria Bacteroidota Bacteroidia Flavobacteriales Flavobacteriaceae ASV5926 ASV5926 | Bacteria Bacteroidota Bacteroidia Chitinophagales Saprospiraceae Aureispira ASV5511 |
|  | Bacteria Bacteroidota Bacteroidia Flavobacteriales Flavobacteriaceae ASV5926 ASV5926 |
| **Microbiota common to nauplii collected on D0 and on D1** | **Microbiota common to nauplii collected on D0 and on D1** |
| Bacteria Bacteroidota Bacteroidia Chitinophagales Saprospiraceae Aureispira ASV48 | Bacteria Bacteroidota Bacteroidia Chitinophagales Saprospiraceae Aureispira ASV48 |
| Bacteria Proteobacteria Alphaproteobacteria Rhodobacterales Rhodobacteraceae Pseudooceanicola ASV60 | Bacteria Proteobacteria Alphaproteobacteria Rhodobacterales Rhodobacteraceae Pseudooceanicola ASV60 |
| Bacteria Proteobacteria Alphaproteobacteria Caulobacterales Hyphomonadaceae Ponticaulis ASV131 | Bacteria Proteobacteria Alphaproteobacteria Caulobacterales Hyphomonadaceae Ponticaulis ASV131 |
| Bacteria Bdellovibrionota Bdellovibrionia Bdellovibrionales Bdellovibrionaceae OM27 clade ASV139 | Bacteria Bdellovibrionota Bdellovibrionia Bdellovibrionales Bdellovibrionaceae OM27 clade ASV139 |
| Bacteria Proteobacteria Gammaproteobacteria Pseudomonadales Cellvibrionaceae Pseudoteredinibacter ASV146 | Bacteria Proteobacteria Gammaproteobacteria Pseudomonadales Cellvibrionaceae Pseudoteredinibacter ASV146 |
| Bacteria Proteobacteria Alphaproteobacteria Caulobacterales Hyphomonadaceae Maricaulis ASV154 | Bacteria Proteobacteria Alphaproteobacteria Caulobacterales Hyphomonadaceae Maricaulis ASV154 |
| Archaea Nanoarchaeota Nanoarchaeia Woesearchaeales SCGC AAA286-E23 ASV158 ASV158 | Archaea Nanoarchaeota Nanoarchaeia Woesearchaeales SCGC AAA286-E23 ASV158 ASV158 |
| Bacteria Bacteroidota Bacteroidia Flavobacteriales Cryomorphaceae Vicingus ASV477 | Bacteria Bacteroidota Bacteroidia Flavobacteriales Cryomorphaceae Vicingus ASV477 |
| Bacteria Proteobacteria Gammaproteobacteria Salinisphaerales Solimonadaceae Oceanococcus ASV681 | Bacteria Proteobacteria Gammaproteobacteria Salinisphaerales Solimonadaceae Oceanococcus ASV681 |
| Bacteria Bacteroidota Bacteroidia Cytophagales Cyclobacteriaceae Reichenbachiella ASV732 | Bacteria Bacteroidota Bacteroidia Cytophagales Cyclobacteriaceae Reichenbachiella ASV732 |
| Bacteria Bacteroidota Bacteroidia Flavobacteriales Cryomorphaceae Vicingus ASV1148 | Bacteria Bacteroidota Bacteroidia Flavobacteriales Cryomorphaceae Vicingus ASV1148 |
| **Nauplii collected on D1** | **Nauplii collected on D1** |
| Bacteria Bacteroidota Bacteroidia Flavobacteriales Flavobacteriaceae Tenacibaculum ASV128 | Bacteria Bacteroidota Bacteroidia Flavobacteriales Flavobacteriaceae Tenacibaculum ASV128 |
| Bacteria Proteobacteria Alphaproteobacteria Rhodobacterales Rhodobacteraceae ASV242 ASV242 | Bacteria Proteobacteria Alphaproteobacteria Rhodobacterales Rhodobacteraceae ASV242 ASV242 |
| Bacteria Firmicutes Bacilli Lactobacillales Lactobacillaceae Leuconostoc ASV306 | Bacteria Firmicutes Bacilli Lactobacillales Lactobacillaceae Leuconostoc ASV306 |
| Bacteria Proteobacteria Gammaproteobacteria Coxiellales Coxiellaceae Coxiella ASV340 | Bacteria Proteobacteria Gammaproteobacteria Coxiellales Coxiellaceae Coxiella ASV340 |
| Bacteria Proteobacteria Alphaproteobacteria Caulobacterales Parvularculaceae ASV429 ASV429 | Bacteria Proteobacteria Alphaproteobacteria Caulobacterales Parvularculaceae ASV429 ASV429 |
| Bacteria Proteobacteria Gammaproteobacteria Pseudomonadales Halieaceae Luminiphilus ASV431 | Bacteria Proteobacteria Gammaproteobacteria Pseudomonadales Halieaceae Luminiphilus ASV431 |
| Bacteria Proteobacteria Alphaproteobacteria Caulobacterales Hyphomonadaceae Ponticaulis ASV472 | Bacteria Proteobacteria Alphaproteobacteria Caulobacterales Hyphomonadaceae Ponticaulis ASV472 |
| Bacteria Dependentiae Babeliae Babeliales UBA12409 ASV493 ASV493 | Bacteria Dependentiae Babeliae Babeliales UBA12409 ASV493 ASV493 |
| Bacteria Proteobacteria Alphaproteobacteria Sphingomonadales Sphingomonadaceae Erythrobacter ASV534 | Bacteria Proteobacteria Alphaproteobacteria Sphingomonadales Sphingomonadaceae Erythrobacter ASV534 |
| Bacteria Bacteroidota Bacteroidia Chitinophagales Saprospiraceae Aureispira ASV555 | Bacteria Bacteroidota Bacteroidia Chitinophagales Saprospiraceae Aureispira ASV555 |
| Bacteria Proteobacteria Alphaproteobacteria Holosporales Holosporaceae ASV1041 ASV1041 | Bacteria Proteobacteria Alphaproteobacteria Holosporales Holosporaceae ASV1041 ASV1041 |
| **Zoea** | **Zoea** |
| Bacteria Bacteroidota Bacteroidia Chitinophagales Saprospiraceae Lewinella ASV79 | Bacteria Bacteroidota Bacteroidia Flavobacteriales Flavobacteriaceae Tenacibaculum ASV76 |
| Bacteria Proteobacteria Gammaproteobacteria Enterobacterales Pseudoalteromonadaceae Pseudoalteromonas ASV184 | Bacteria Bacteroidota Bacteroidia Cytophagales Microscillaceae Microscilla ASV95 |
|  | Bacteria Bacteroidota Bacteroidia Cytophagales Bernardetiaceae Bernardetia ASV199 |
| **Zoea-Mysis** | **Zoea-Mysis** |
| Bacteria Proteobacteria Alphaproteobacteria Rhodobacterales Rhodobacteraceae Litorimicrobium ASV161 |  |
| **Mysis** | **Mysis** |
| Bacteria Proteobacteria Gammaproteobacteria Thiotrichales Thiotrichaceae Thiothrix ASV32 | Bacteria Proteobacteria Gammaproteobacteria Thiotrichales Thiotrichaceae Thiothrix ASV32 |
| Bacteria Proteobacteria Alphaproteobacteria Micavibrionales Micavibrionaceae ASV42 ASV42 | Bacteria Proteobacteria Alphaproteobacteria Micavibrionales Micavibrionaceae ASV42 ASV42 |
| Bacteria Bacteroidota Bacteroidia Chitinophagales Saprospiraceae Phaeodactylibacter ASV65 | Bacteria Bacteroidota Bacteroidia Chitinophagales Saprospiraceae Phaeodactylibacter ASV65 |
| Bacteria Proteobacteria Alphaproteobacteria Rhodobacterales Rhodobacteraceae Ruegeria ASV93 | Bacteria Proteobacteria Gammaproteobacteria Enterobacterales Pseudoalteromonadaceae Pseudoalteromonas ASV83 |
| Bacteria Planctomycetota Phycisphaerae Phycisphaerales Phycisphaeraceae SM1A02 ASV100 | Bacteria Proteobacteria Gammaproteobacteria Pseudomonadales Saccharospirillaceae Thalassolituus ASV89 |
| Bacteria Proteobacteria Gammaproteobacteria Enterobacterales Vibrionaceae Vibrio ASV118 | Bacteria Proteobacteria Gammaproteobacteria Enterobacterales Vibrionaceae Vibrio ASV118 |
| Bacteria Bacteroidota Bacteroidia Flavobacteriales Flavobacteriaceae Winogradskyella ASV132 | Bacteria Bacteroidota Bacteroidia Flavobacteriales Flavobacteriaceae ASV175 ASV175 |
| Bacteria Bacteroidota Bacteroidia Flavobacteriales Cryomorphaceae Vicingus ASV162 |  |
| Bacteria Bacteroidota Bacteroidia Flavobacteriales Flavobacteriaceae ASV175 ASV175 |  |
| Bacteria Planctomycetota Planctomycetes Planctomycetales Rubinisphaeraceae ASV225 ASV225 |  |
| Bacteria Proteobacteria Alphaproteobacteria Kiloniellales Kiloniellaceae Kiloniella ASV228 |  |
| Bacteria Bacteroidota Bacteroidia Flavobacteriales Flavobacteriaceae Mesoflavibacter ASV300 |  |
| Bacteria Proteobacteria Gammaproteobacteria Pseudomonadales Cellvibrionaceae Pseudoteredinibacter ASV381 |  |
| **Core microbiota** | **Core microbiota** |
| Bacteria Proteobacteria Gammaproteobacteria Enterobacterales Vibrionaceae Vibrio ASV1 | Bacteria Proteobacteria Gammaproteobacteria Enterobacterales Vibrionaceae Vibrio ASV1 |
| Bacteria Proteobacteria Gammaproteobacteria Enterobacterales Pseudoalteromonadaceae Pseudoalteromonas ASV2 | Bacteria Proteobacteria Gammaproteobacteria Enterobacterales Pseudoalteromonadaceae Pseudoalteromonas ASV2 |
| Bacteria Proteobacteria Gammaproteobacteria Enterobacterales Pseudoalteromonadaceae Pseudoalteromonas ASV3 | Bacteria Proteobacteria Gammaproteobacteria Enterobacterales Pseudoalteromonadaceae Pseudoalteromonas ASV3 |
| Bacteria Proteobacteria Gammaproteobacteria Enterobacterales Alteromonadaceae Alteromonas ASV4 | Bacteria Proteobacteria Gammaproteobacteria Enterobacterales Alteromonadaceae Alteromonas ASV4 |
| Bacteria Proteobacteria Gammaproteobacteria Pseudomonadales Saccharospirillaceae Thalassolituus ASV5 | Bacteria Proteobacteria Gammaproteobacteria Pseudomonadales Saccharospirillaceae Thalassolituus ASV5 |
| Bacteria Proteobacteria Gammaproteobacteria Pseudomonadales Cellvibrionaceae Pseudoteredinibacter ASV6 | Bacteria Proteobacteria Gammaproteobacteria Pseudomonadales Cellvibrionaceae Pseudoteredinibacter ASV6 |
| Bacteria Proteobacteria Gammaproteobacteria Enterobacterales Alteromonadaceae Aestuariibacter ASV7 | Bacteria Proteobacteria Gammaproteobacteria Enterobacterales Alteromonadaceae Aestuariibacter ASV7 |
| Bacteria Proteobacteria Alphaproteobacteria Rhodobacterales Rhodobacteraceae Shimia ASV9 | Bacteria Proteobacteria Alphaproteobacteria Rhodobacterales Rhodobacteraceae Shimia ASV9 |
| Bacteria Proteobacteria Alphaproteobacteria Rhodobacterales Rhodobacteraceae Nautella ASV10 | Bacteria Proteobacteria Alphaproteobacteria Rhodobacterales Rhodobacteraceae Nautella ASV10 |
| Bacteria Bacteroidota Bacteroidia Chitinophagales Saprospiraceae ASV11 ASV11 | Bacteria Bacteroidota Bacteroidia Chitinophagales Saprospiraceae ASV11 ASV11 |
| Bacteria Proteobacteria Alphaproteobacteria Rhodobacterales Rhodobacteraceae Roseobacter clade CHAB-I-5 lineage ASV12 | Bacteria Proteobacteria Alphaproteobacteria Rhodobacterales Rhodobacteraceae Roseobacter clade CHAB-I-5 lineage ASV12 |
| Bacteria Proteobacteria Gammaproteobacteria | Bacteria Proteobacteria Gammaproteobacteria |
| Enterobacterales Vibrionaceae Vibrio ASV14 | Enterobacterales Vibrionaceae Vibrio ASV14 |
| Bacteria Proteobacteria Gammaproteobacteria Pseudomonadales Marinobacteraceae Marinobacter | Bacteria Proteobacteria Gammaproteobacteria Pseudomonadales Marinobacteraceae Marinobacter ASV15 |
| ASV15 |  |
| Bacteria Proteobacteria Gammaproteobacteria Pseudomonadales Oleiphilaceae Oleiphilus ASV16 | Bacteria Proteobacteria Gammaproteobacteria Pseudomonadales Oleiphilaceae Oleiphilus ASV16 |
| Bacteria Proteobacteria Alphaproteobacteria Caulobacterales Hyphomonadaceae Hyphomonas ASV17 | Bacteria Proteobacteria Alphaproteobacteria Caulobacterales Hyphomonadaceae Hyphomonas ASV17 |
| Bacteria Proteobacteria Gammaproteobacteria Enterobacterales Pseudoalteromonadaceae Pseudoalteromonas ASV21 | Bacteria Proteobacteria Gammaproteobacteria Enterobacterales Pseudoalteromonadaceae Pseudoalteromonas ASV21 |
| Bacteria Bacteroidota Bacteroidia Chitinophagales Saprospiraceae Lewinella ASV23 | Bacteria Bacteroidota Bacteroidia Chitinophagales Saprospiraceae Lewinella ASV23 |
| Bacteria Proteobacteria Gammaproteobacteria Enterobacterales Idiomarinaceae Idiomarina ASV26 | Bacteria Proteobacteria Gammaproteobacteria Enterobacterales Idiomarinaceae Idiomarina ASV26 |
| Bacteria Proteobacteria Gammaproteobacteria Pseudomonadales Halomonadaceae Halomonas ASV30 | Bacteria Proteobacteria Gammaproteobacteria Enterobacterales Colwelliaceae Thalassotalea ASV34 |
| Bacteria Proteobacteria Gammaproteobacteria Enterobacterales Colwelliaceae Thalassotalea ASV34 | Bacteria Bacteroidota Bacteroidia Cytophagales Cyclobacteriaceae Fabibacter ASV36 |
| Bacteria Bacteroidota Bacteroidia Cytophagales Cyclobacteriaceae Fabibacter ASV36 | Bacteria Proteobacteria Alphaproteobacteria Rhodobacterales Rhodobacteraceae Nautella ASV38 |
| Bacteria Bacteroidota Bacteroidia Chitinophagales Saprospiraceae Aureispira ASV37 | Bacteria Proteobacteria Alphaproteobacteria Caulobacterales Hyphomonadaceae Hyphomonas ASV40 |
| Bacteria Proteobacteria Alphaproteobacteria Rhodobacterales Rhodobacteraceae Nautella ASV38 | Bacteria Proteobacteria Alphaproteobacteria Rhodobacterales Rhodobacteraceae Ruegeria ASV41 |
| Bacteria Proteobacteria Alphaproteobacteria Caulobacterales Hyphomonadaceae Hyphomonas ASV40 | Bacteria Proteobacteria Alphaproteobacteria Rhodobacterales Rhodobacteraceae Leisingera ASV44 |
| Bacteria Proteobacteria Alphaproteobacteria Rhodobacterales Rhodobacteraceae Ruegeria ASV41 | Bacteria Bacteroidota Bacteroidia Flavobacteriales Cryomorphaceae Owenweeksia ASV45 |
| Bacteria Proteobacteria Alphaproteobacteria Rhodobacterales Rhodobacteraceae Leisingera ASV44 | Bacteria Proteobacteria Alphaproteobacteria Rhodospirillales Terasakiellaceae ASV62 ASV62 |
| Bacteria Bacteroidota Bacteroidia Flavobacteriales Cryomorphaceae Owenweeksia ASV45 | Bacteria Proteobacteria Alphaproteobacteria Rhodobacterales Rhodobacteraceae Epibacterium ASV69 |
| Bacteria Proteobacteria Alphaproteobacteria Rhodospirillales Terasakiellaceae ASV62 ASV62 | Bacteria Proteobacteria Gammaproteobacteria Enterobacterales Colwelliaceae Thalassotalea ASV78 |
| Bacteria Firmicutes Bacilli Staphylococcales Staphylococcaceae Staphylococcus ASV123 | Bacteria Proteobacteria Gammaproteobacteria Gammaproteobacteria Incertae Sedis Unknown Family Marinicella ASV127 |
| Bacteria Proteobacteria Gammaproteobacteria Gammaproteobacteria Incertae Sedis Unknown Family Marinicella ASV127 |  |
